# The impact of control and mitigation strategies during the second wave of coronavirus infections in Spain and Italy

**DOI:** 10.1038/s41598-022-05041-0

**Published:** 2022-01-20

**Authors:** Marco De Nadai, Kristof Roomp, Bruno Lepri, Nuria Oliver

**Affiliations:** 1 Fondazione Bruno Kessler (FBK), Povo, Italy; 2 ELLIS Unit Alicante Foundation, Alicante, Spain; 3 Microsoft-United States, Redmond, USA

**Keywords:** Health policy, Epidemiology, Viral infection

## Abstract

European countries struggled to fight against the second and the third waves of the COVID-19 pandemic, as the Test-Trace-Isolate (TTI) strategy widely adopted over the summer and early fall 2020 failed to contain the spread of the disease effectively. This paper sheds light on the effectiveness of such a strategy in two European countries (Spain and Italy) by analysing data from June to December 2020, collected via a large-scale online citizen survey with 95,251 and 43,393 answers in Spain and Italy, respectively. Our analysis describes several weaknesses in each of the three pillars of the TTI strategy: Test, Trace, and Isolate. We find that 40% of respondents had to wait more than 48 hours to obtain coronavirus tests results, while literature has shown that a delay of more than one day might make tracing all cases inefficient. We also identify limitations in the manual contact tracing capabilities in both countries, as only 29% of respondents in close contact with a confirmed infected individual reported having been contact traced. Moreover, our analysis shows that more than 45% of respondents report being unable to self-isolate if needed. We also analyse the mitigation strategies deployed to contain the second wave of coronavirus. We find that these interventions were particularly effective in Italy, where close contacts were reduced by more than 20% in the general population. Finally, we analyse the participants’ perceptions about the coronavirus risk associated with different daily activities. We observe that they are often gender- and age-dependent, and not aligned with the actual risk identified by the literature. This finding emphasises the importance of deploying public-health communication campaigns to debunk misconceptions about SARS-CoV-2. Overall, our work illustrates the value of online citizen surveys to quickly and efficiently collect large-scale population data to support and evaluate policy decisions to combat the spread of infectious diseases, such as coronavirus.

## Introduction

Since March 2020, Europe has been fighting the coronavirus pandemic. The first wave of infections during March and April 2020 led to a significant excess in mortality rates^1–4^ and caused unprecedented pressure in the healthcare systems of many European countries. After applying severe confinement measures from mid-March until June 2020, the first wave of SARS-CoV-2 infections was controlled in Europe^5–7^. As a result, companies and retail activities reopened, and the majority of non-pharmaceutical interventions were lifted^8,9^. To prevent a potential second wave of infections, most of the European governments focused on a control strategy called Test-Trace-Isolate (TTI)^10–13^, aimed at detecting and isolating all infected individuals as early as possible to limit their ability to infect other people and thus reduce the number of coronavirus cases.

Previous works have shown that TTI can effectively contain the spread of SARS-CoV-2 if physical distancing measures are implemented and complied with^10–14^. However, applying an effective TTI strategy is far from easy. For example, testing should be as fast as possible, such that a delay of 3 days or more might make tracing inefficient^15^. Similarly, scholars found that contact tracing should cover at least 50% of contacts and possibly be helped by digital contact tracing via apps with more than 20% of adoption^15^. Moreover, the availability of isolation infrastructure has been highlighted as a critical element for the TTI strategy to succeed^16^. In sum, previous work has pointed out several crucial conditions that would need to be met for TTI to work, not to mention the role played by pre-symptomatic and asymptomatic individuals in the spread of coronavirus^17–21^ that challenges the overall TTI strategy. Furthermore, the three pillars in TTI are interrelated, having a cascading impact on the others. While TTI has been successfully used to prevent new outbreaks in different countries^22,23^, the emergence of second and third waves of coronavirus infections in Europe poses questions about the efficacy of the implemented TTI control strategies. Unfortunately, governments do not typically collect (or are unable to share) detailed statistics about each of the three pillars—namely, Test-Trace-Isolate—of this strategy, making it challenging to identify the strengths and weaknesses of the implemented measures to control the spread of coronavirus.

This manuscript describes an analysis of the effectiveness of the pandemic control strategies and mitigation interventions implemented in Spain and Italy between June and December of 2020. We analyse data collected via a large-scale online citizen survey with 95,251 answers in Spain and 43,393 answers in Italy. We focus on Spain and Italy as they have been two of the most affected European countries with more than 65,000 reported COVID-19 cases and over 1,660 COVID-19 related deaths per million people as of May 2021^24^. Both countries share similar cultures yet exhibited a different pattern in the emergence of the second wave of coronavirus cases: in Spain, the number of coronavirus infections started increasing in mid-July of 2020, leading to a long second wave of infections which reached its peak in the first week of November of 2020. On the other hand, in mid-July of 2020, the number of confirmed coronavirus cases in Italy was still very low, leading the Spanish press to theorise that Italy might have been doing significantly better than Spain in controlling the growth of coronavirus infections via their TTI strategy. Our analysis aims to understand the potential factors that could have contributed to this differential pandemic spread in the summer and fall of 2020 between the two countries. Thus, we divide our analysis into two phases: a first phase between the end of the first wave and the start of the second wave of coronavirus infections (between June and September 2020) and a second phase, corresponding to the second wave of SARS-CoV-2 infections (between October and December 2020).

Through our analysis, we identify several weaknesses in each of the three pillars of the TTI control strategy. Moreover, we shed light on the participants’ behaviour before and after the mitigation strategies were deployed during the second wave of infections. Our work illustrates the value of online surveys as a cheap and efficient tool to quickly collect large-scale population data about people’s perception, situation and self-reported behaviour during a pandemic.

## Results

We analyse 138,644 answers to an anonymous online survey called COVID19ImpactSurvey^25^, which two of the authors designed and launched on March 28th 2020. The survey consists of 26 questions (some were added or removed over time in order to stay relevant to the changing situation) that ask participants about their demographic and household information, their social behaviour and adopted protection measures to prevent a coronavirus infection (e.g. face mask usage), their ability to isolate, their willingness to get tested and vaccinated, their perception about the adopted government measures and the risk of infection associated with different activities/places (e.g. restaurants). Our results are based on analysing a subset of 24 questions of this survey as per Table S1 in Supplementary Information (SI). We focus on the answers collected between the end of June and the end of December 2020 in Spain and Italy, namely 95,251 and 43,393 answers, respectively. Each day, the survey collected, on average, 491 and 217 anonymous answers in Spain and Italy, respectively. Users were required to be at least 18 years old.

The gender and age distributions of the collected sample are not proportional to those of the general population of Spain and Italy. Thus, we follow the methodology described in Oliver et al.^25^ and re-weight the answers such that the resulting gender and age distributions match the official statistics of each NUTS-3 statistical region^26^ in Spain (i.e., provinces, islands, and the autonomous cities of Ceuta and Melilla) and Italy (i.e., provinces) in 2020. We also filter entries with inconsistent answers (4% and 6% of answers in Spain and Italy, respectively) and entries that appear to have been answered too fast (2% and 4% of answers in Spain and Italy, respectively) or too slow (2% and 3% of answers in Spain and Italy, respectively). In total, we discard 8% and 13% of the answers in Spain and Italy, respectively. We refer to the Methodology section for additional details. All answers are categorical or binary, thus we report the percentage of participants who selected each response and compute the 95% confidence intervals through the margin of error.

We temporally divide our analyses according to two phases: (1) *Phase I—new normality* and (2) *Phase II—second wave*. *Phase I—new normality* corresponds to the time period between June and October 2020 (Italy: July 31st–October 26th, Spain: June 21st–October 25th). During this phase, both Spain and Italy lifted most of the non-pharmaceutical interventions deployed since March 2020 and only imposed local measures. Hence, this period is referred to as the *new normality*. Most of the activities were re-established as they were before the start of the pandemic except for requiring facial mask wearing in indoor/outdoor public spaces, defining limitations on large gatherings and light-weight restrictions such on the occupancy of gyms, restaurants, theatres and cinemas. *Phase II—second wave* corresponds to the period between October and December 2020 (Italy: October 26th–December 31st, Spain: October 25th–December 31st), when the second wave of the coronavirus pandemic took place in both countries. During this phase, the countries implemented a range of non-pharmaceutical interventions to contain the spread of the disease, including mobility restrictions, partial closings of restaurants, bars, coffee shops and gyms, and cancellations or limits on the size of large events (e.g. sports events, weddings, funerals) and on the number of people in small family/social gatherings. We refer to the Methodology Section for additional details.

We first report our findings through the lens of the survey related to the Test-Trace-Isolate control strategies implemented in Spain and Italy during the *new normality* phase, followed by an analysis of the reported changes in the respondents’ behaviours as a consequence of the implemented mitigation interventions deployed during the *second wave* phase.

### Effectiveness of the test, trace, and isolate control strategies in Spain and Italy

**Figure 1:**
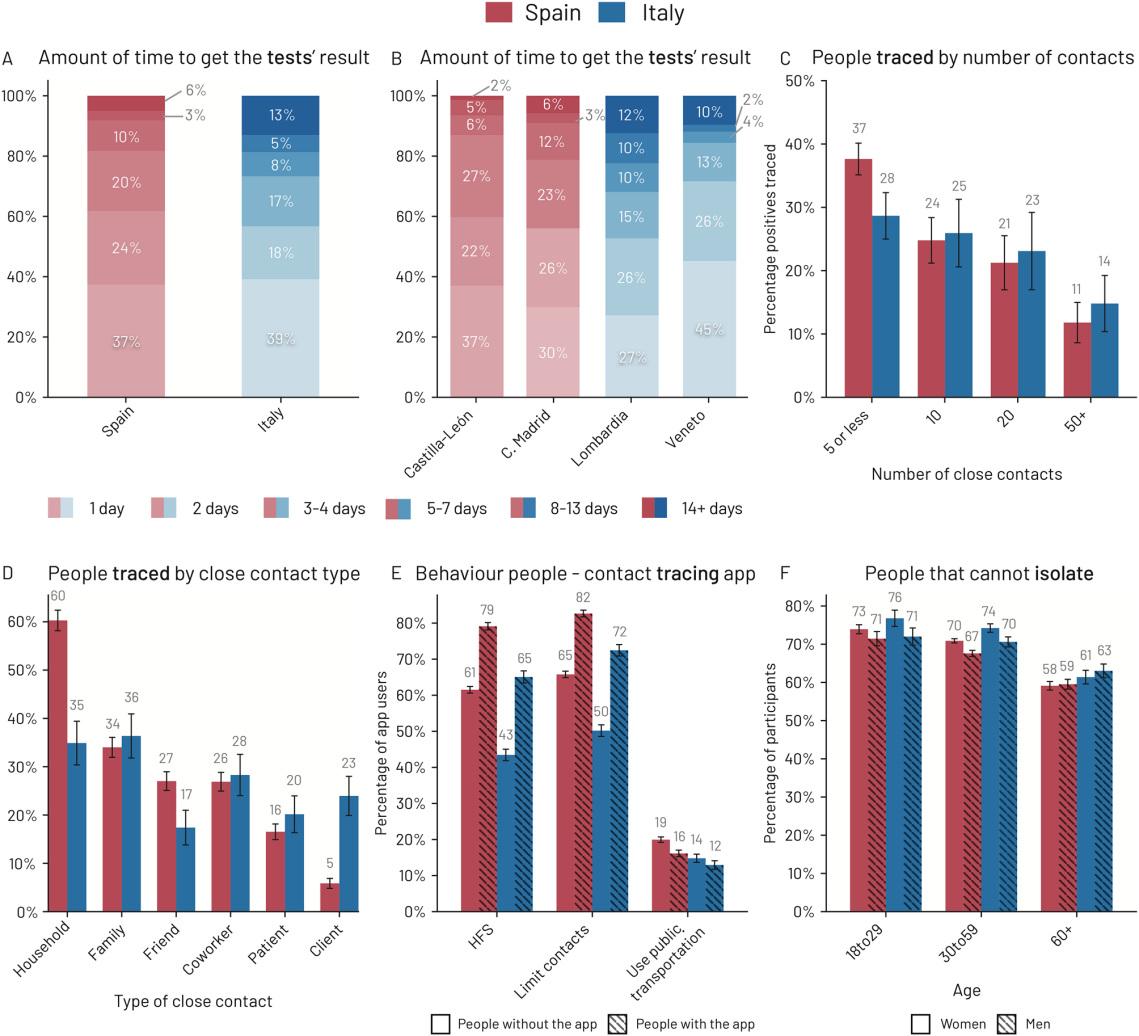
The test-trace-isolate (TTI) control strategy during the Phase I—*new normality* in Spain (red) and Italy (blue). **(A)** Amount of time to get the results of a COVID-19 test in Spain and Italy; **(B)** amount of time to get the results of a COVID-19 test in two exemplar regions for Spain and in two exemplar regions for Italy; **(C)** percentage of close contacts that were contact-traced by the health authority in Spain and Italy. The majority of respondents who had a close contact with a positive case report were not contacted by the health authority. **(D)** Percentage of close contacts traced for different categories of contacts in Spain and Italy. Spain focused the tracing activity within the household, where 60% of the people reported having been contact traced. **(E)** Protection measures adopted by people in Spain and Italy, divided by whether they installed the contact tracing app. Respondents who installed the contact tracing apps are more likely to report complying with the washing Hands, Face masks, making Space (HFS) recommendations, limit their close contacts and using less public transportations. **(F)** Percentage of people, in Spain and Italy, who report being unable to isolate effectively if needed. In plots **(C–F)** we report the 95% confidence intervals.

#### Test

The first TTI pillar entails promptly testing all suspected individuals with a coronavirus infection due to having had close contact with an confirmed infected case and/or exhibiting symptoms attributable to COVID-19.

Figure 1A shows that 61% and 57% of respondents, in Spain and Italy respectively, reported receiving the test results within 48 hours from taking the test (Q24 answers a-b-d-f and Q24_3 in SI Table S1). However, we observe remarkable differences per administrative region. In the Autonomous Community of Madrid (Spain) 56% of tests took 48 h and 9% of tests took 8 days or more, while in Castilla-Leon (Spain) 59% of tests were reported within 48 hours and only 7% took eight days or more. Similarly, in Lombardia (Italy) 53% of tests took 48 h to be reported and 22% of the tests took 8 days or more. Veneto (Italy) was more efficient than Lombardia: only 12% of the tests required 8 days or more to be processed (see Fig. 1B).

In addition, we have evidence that the time required to get a test result decreased over time in Spain and Italy. In Italy, the average waiting time to receive the test results in the week number 41 (October 5–11, 2020) was $4.0\pm 0.5$ days, while in week 51 (December 14–20, 2020) it went down to around $2.9\pm 0.2$ days. Similarly, in Spain, it changed from $2.8\pm 0.2$ days in week 41 to around $2.6\pm 0.2$ days by the end of December (see SI Fig. S7). This decrease in waiting time is also confirmed in Italy by the official data shared by the Italian health authority^27^. Note that the shared data might be noisy and influenced by how it is reported to the health authority^28^. Thus, it is not possible to compare the absolute values between our survey and the official data. However, we observe that over time, Italian delays in reporting COVID-19 test results seem to decrease from 7.5 days on average in September 2020 to around three days at the end of December 2020. As expected, we see a weekly trend where there are fewer tests on the weekends and holidays. We refer to SI Fig. S8 for additional details.

#### Trace

Contact tracing is typically performed by case investigators and contact tracers, specifically trained to locate people who have tested positive for coronavirus and talk with them. The focus of contact tracing is understanding how people got infected, identifying the close contacts they had up to that point and determining which of those should be contacted to recommend testing and isolation if necessary.

To estimate the level of contact tracing implemented in each country, we analyse the responses of participants who report having had a close contact with someone infected by COVID-19 (Q11 in SI Table S1). Following the World Health Organization’s definition, a close contact is someone who has been within 1.5 meters of an infected individual for at least 15 cumulative minutes over a 24-hour period^29^. From those, we determine the percentage who also report having been called by a contact tracer (Q11_1 in SI Table S1).

Regarding the percentage of those who reported having had a close contact with a positive individual and were contact traced, we obtain similar results in Spain and Italy. We find that only $29\%$ of respondents reported having been called by the contact tracers (Italy: 25%, Spain: $33\%$). Figure 1C shows the results of the two countries aggregated by the number of weekly contacts (Q9 in SI Table S1). Interestingly, we observe that the two countries were especially weak in tracing participants with the highest number of close contacts (50+). We also investigate contact tracing among those who reported having tested positive for coronavirus when filling out the survey (Q24 answers a–b–d–f and Q24_3 in SI Table S1). We find that 75% of participants reported having been contacted by contact tracers (Italy: $78\%$, Spain: $74\%$); 62% declared that some of their close contacts were traced (Italy: $62\%$, Spain: $62\%$), and 42% reported that their close contacts were also tested for coronavirus (Italy: $33\%$, Spain: $46\%$) (see SI Fig. S11).

We observe some differences between Spain and Italy when we consider the *type of contact* (Q9_1 in SI Table S1) traced. According to our data, coworkers and friends are the most common types of close contacts reported by infected individuals (see SI Fig. S9). However, Fig. 1D shows that less than 25% of these contacts were traced, while the contact types that were most frequently traced are within the household and family. Notably, in 60% of the cases, the household contacts were contact traced in Spain, while the other types of the close contacts were traced less than 40% of the time.

We also computed the trend of the percentage of participants who reported having had a close contact with a positive case and having been traced by the health authority in Italy. We fit this data through a symmetric Locally Estimated Scatterplot Smoothing (LOESS) model^30^ and verify the fit is robust to outliers (see SI Section S4 for additional details). SI Fig. S7 illustrates that, in Italy, the percentage of traced contacts per infected individual declined as the number of positive COVID-19 cases started to reach its all-time high, suggesting a saturation of the contact tracing capacity. We report additional details of this analysis in the Methodology section.

To complement manual contact tracing efforts, many governments in the world launched smartphone apps to automatically trace and inform people who had been in close contact with an infected individual, following the theoretical model proposed by Ferretti et al.^31^. Spain and Italy launched in the summer of 2020 their Bluetooth-based digital contact tracing apps based on the GAEN (Google and Apple Exposure Notification) interface^32^: RadarCOVID launched on August 14th 2020 in Spain, and Immuni launched on June 15th 2020 in Italy. While the apps had reached 6.3 and 10.1 million users respectively by the end of December 2020, their penetration was not evenly distributed demographically. According to our data, in Italy, $50\%$ of male respondents aged 18–29 years old, $41\%$ of male respondents aged 60+ years and $47\%$ of female respondents aged 60+ reported having the app installed (Q20 in SI Table S1). These figures are higher than the officially reported adoption statistics for Italy, reflecting a technology bias in our sample. In Spain, we identify gender and age differences: women do not install the app as much as men do, and younger participants are less likely to have the app installed than older individuals. We refer to SI Fig. S12 for additional details.

Interestingly, we observe that app users adopt more countermeasures to prevent the spread of coronavirus than non-app users (Q20 in SI Table S1). Figure 1E shows that app users are more likely than non-app users (71% vs 53%) to comply with the Hands/Face/Space (HFS) recommended measures—i.e. wash/disinfect their hands, wear face masks and maintain physical distance with other people; they tend to limit their close contacts more (77% vs 58%) and use less frequently public transportation to commute (14% vs 17%). We refer to SI Fig. S16 for additional details.

Our data also allows us to understand the added value of the apps to trace contacts with positive individuals (Q11 and Q11_1 in SI Table S1). We observe that only 2% of respondents who reported having had a close contact with an infected individual received a notification from the app (Italy: 3%, Spain: 1%). Moreover, only 1% of respondents (Italy: 2%, Spain: less than 1%) had close contact with a positive case discovered through the app. Of those, only one case in Italy got tested, while none of them got tested in Spain out of the 698 people who got a notification, according to the survey (Q11_1 in SI Table S1).

#### Isolate

Typically, European governments ask individuals with confirmed coronavirus infections to self-isolate at home^16^, in addition to those with COVID-19 compatible symptoms and those who had close contact with an infected individual until obtaining their coronavirus test results. However, we find that a non-negligible percentage of respondents report being unable to self-isolate due to various factors (Q19 in SI Table S1). Figure 1F shows that more than 54% of participants aged 18–59 years old respond being unable to isolate effectively. Fortunately, older (60+ years old) and more vulnerable participants report having fewer self-isolation problems (less than 38% report being unable to self isolate). Our data also shows that more than 7% of respondents would have financial difficulties (Italy: 9%, Spain: 7%), are afraid of stigmatisation (Italy: 11%, Spain: 7%), and/or find isolation psychologically impossible (Italy: 10%, Spain: 8%). Moreover, we identify age and gender differences in the reported barriers. Women aged 30 to 59 years old are the most likely demographic group to report an inability to self-isolate due to having to take care of children (29% in Italy and 28% in Spain). Interestingly, men of the same age group are less likely to report facing the same barrier (14% in Italy and 13% in Spain, Welch-t test: 37.11 p-value<0.001). Similar differences are present between women aged 18 to 29 (10% in Italy and 13% in Spain) and men aged 18 to 29 (4% in Italy and Spain) years old (Welch-t test: 14.98 p-value<0.001).

The youth (aged 18–20 years old, and particularly women) is the most likely demographic group to report psychological barriers to self-isolation: 22% of youth in Italy and 19% of youth in Spain report being afraid of stigmatisation due to coronavirus; and 28% of youth in Italy and 21% of youth in Spain report not being able to self-isolate due to psychological factors. More than 40% of Italian women aged 18 to 20 years old declare that it would be psychologically impossible for them to be quarantined for at least ten days, while more than 24% are afraid of stigmatisation. Similar results apply to young respondents in Spain.

Financial and labour barriers that prevent self-isolation are the most frequent among those aged 21 to 39, with notable differences compared to other age groups. Conversely, the elderlies (aged 60+) are the most likely demographic group to report being able to self-isolate. For example, in Italy, 18% of the elderly vs 35% for those aged 18-59 years old report not being able to self isolate. Comparable results are obtained for Spain. We refer to the SI Figure S13 for additional details.

## Mitigation interventions

In this section, we compare several aspects of the participants’ self-reported behaviour before and after the non-pharmaceutical interventions were deployed to mitigate the second wave of coronavirus infections in each country, namely: the estimated number of weekly close contacts from outside the household, the perception of risk associated with daily life activities and places, and the type of socialisation environment.

### Number of weekly close contacts from outside the household

With the rise of confirmed coronavirus cases in Italy in early October, the Italian Government imposed gradually stricter rules from the compulsory use of face masks in public spaces on October 7th 2020, to the definition of a three-tiered system on November 6th 2020^33^. This system defined restrictions based on the level of transmission of the virus.

**Figure 2:**
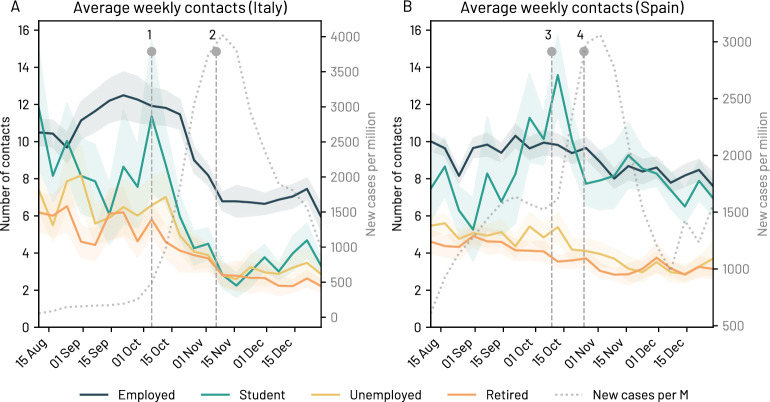
Average estimated number of weekly close contacts from outside the household together with the number of daily coronavirus infections (dotted curve) in Italy **(A)** and Spain **(B)**. We highlight in the plots several notable events: (1) the Italian compulsory mask mandate in public spaces; (2) the imposition of the three-tiered system in Italy; (3) the Spanish declaration of state of alarm in the Comunidad de Madrid; and (4) the declaration of state of emergency in Spain. We report the 95% confidence interval to ease the comparison of the trends.

Our data suggest that these measures effectively reduced the number of weekly close contacts and the presence at work, and increased the adoption of personal protection measures. We estimate the number of different contacts from outside home that participants reported having in 1 week by averaging the lower bound of the specified bins in the number of contacts (e.g. “3–4”, “5–9”) (Q9 in SI Table S1) to account for the best case scenario. Figure 2A shows a drastic reduction in the estimated number of close contacts from outside the home for the different categories of respondents (students, employed, unemployed and retired). On November 10th, we observe an average reduction of 45% in the number of different close contacts than those reported the week of October 13th. This decrease is particularly large among students and the employed. By November 10th (one week after the implementation of the three-tiered system), the reported number of close contacts was reduced by 81%, 35%, 65% and 55% for students, employed, non-working and retired people, respectively, when compared to September (see Fig. 2A,B). The reduction in the number of close contacts reported by students can be attributed to the restrictions on high schools and universities, which affected their daily number of contacts. When we average the number of close contacts in September and October versus the average number of close contacts from November to the end of December, respondents aged 18 to 20 years old limited their close contacts the most (−58%, from $10.3 \pm 1.2$ to $4.3 \pm 0.3$), followed by those aged 70 to 79 (−49%, from $5.35 \pm 0.7$ to $2.7 \pm 0.2$) and 21 to 29 (−46%, from $9.9 \pm 0.4$ to $5.3 \pm 0.5$) years old. This last group, though, is the group with the smallest number of close contacts from outside the household. We refer to the SI Section S5.1 and Table S2 for additional details on the age range of participants. Employed respondents increased remote working by 9% on average, thus reducing their close contacts at work. We observe an increase both in unemployment (from 6 to 8%) and in those who reported being on unpaid leave (from 2 to 4%). We refer to the SI Table S5, S6 and S7 for additional details.

Before October 2020, Spain did not have a centralised COVID-19 mitigation strategy, leaving to each of the 17 autonomous regions the decision on imposing restrictions as needed. From October 2020, all the autonomous regions in Spain (except for Madrid and the Basque Country) decided to adopt a 5-level risk system based on their healthcare occupancy levels, the cumulative incidence, the positivity rate and the percentage of traced cases. On October 25th, 2020 the government declared again a state of alarm^34^, which enabled establishing a national curfew and gave power to local authorities to ban travel across regions, provinces and municipalities if needed.

Together with the increase in the number of confirmed coronavirus cases, we find that these measures had a gradual and constant effect on the average number of close contacts participants reported having over time. For example, the average number of close contacts from outside the home for students and employed respondents decreased from more than ten in mid-September to around eight at the end of December. When we compare the average number of close contacts from outside the household in one week in the period between September and the end of October with those in the period between November and the end of December, respondents between 18 to 20 years old lowered their close contacts the most (−25%, from $11.5 \pm 1.5$ to $8.3 \pm 0.6$), followed by those aged 50 to 59 (−19%, from $8.3 \pm 0.4$ to $6.7 \pm 0.5$) and 30 to 39 (−18%, from $8.9 \pm 0.3$ to $7.2 \pm 0.4$) years old. These reductions in the number of close contacts are smaller than in Italy. We refer to the SI Section S5.1 and Table S2 for additional details. Surprisingly, teleworking decreased by 35%, from 15% of teleworking in the new normality phase to 10% after the measures were implemented. The percentage of respondents reporting being on unpaid leave decreased by 52% (from 3 to 2%), while those who reported having lost their job rose by 29% (from 8 to 11%).

We quantified the reduction in the number of close contacts with a Theil-Sen regression estimator^35^. We find that the Italian decrease in the number of contacts is remarkably steeper. For example, Italian students experienced a marked reduction in the number of contacts (the estimated slope is −0.39), while Spanish students almost did not reduce them (slope around −0.01). We refer to SI Section S5.2 for additional details.

### Perception of risk of coronavirus infection associated with daily life activities and places

The increase in the number of coronavirus confirmed cases, the measures implemented by the governments and the media possibly contributed to a large change in people’s perception of the risk associated with different places/activities and in the personal protection measures they adopted to minimise their risk of being infected with coronavirus. For example, in July 2020, over 60% of Italian respondents considered that going to the hairdresser entailed a low risk of getting a coronavirus infection. However, this percentage consistently decreased in the fall and winter of 2020, reaching 42% by the end of December 2020. Similarly, in Spain, the perception of safety when buying in small stores decreased by almost 20 percentage points in the same period. We also note a decrease of more than ten percentage points in both countries in the perception of how safe hospitals are, which could have negative consequences if many people chose not to go to the hospital when they should have gone for fear of getting infected with coronavirus. See SI Figs.S14 and S15 for additional information. In the SI we also discuss the implementation of self-protection measures among the population over time.

**Figure 3:**
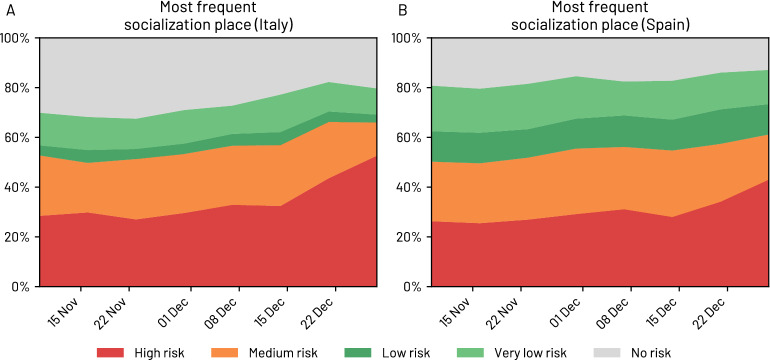
Associated risk of the most frequent place of socialisation for Italy **(A)** and Spain **(B)** between November 1st 2020 and December 31st 2020. Note how socialisation in high-risk environments (i.e. indoors without any protection measures) increases over time (red area in the figure).

### Type of socialisation environment

One of the primary goals of the governments’ pandemic mitigation strategies was limiting the amount of physical contact within the population. However, not all encounters are alike. In the survey, one question asked participants to report the most common type of context where they had socialised with people from outside of their household in the last week (Q10 in SI Table S1). We categorised the answers depending on their risk of infection, according to five levels of risk: *High risk* for indoor private environments (e.g. homes, private clubs) without adopting protection (HFS) measures; Restaurants and bars (indoors), schools and workplaces are considered to be of *Medium risk* as HFS measures are compulsory in such places; Restaurants and bars (outdoors) and other outdoor gathering activities would be labelled as *Low Risk*; Nature, beaches and the street are considered to be *Very low risk* environments; and no social contacts outside of the household would be placed in a *No risk* category.

Figure 3A,B show the associated risk of the most frequent socialisation environment over time for Italy and Spain. Interestingly, we observe a gradual increase in the risk level, as the percentage of those who report zero socialisation decreased and the percentage of people who reported socialising mainly in high-risk environments increased, probably due to the Christmas festivities in Italy and Spain. While the estimated number of weekly close contacts did not increase (Fig. 2A,B), the types of environments where such contacts took place changed over time, with an increase in the prevalence of high-risk environments (Fig. 3A,B). See SI Fig. S19 for a breakdown of the two countries.

## Discussion and implications

In this paper, we have quantitatively analysed and compared the effectiveness of the TTI strategies deployed in Spain and Italy by analysing data collected using a large-scale online survey from June to December 2020. We have also studied the impact of the pandemic mitigation interventions on the respondents’ self-reported behaviour and perceptions, namely the estimated number of distinct close contacts from outside the home, the perception of risk of coronavirus infection associated with common activities and places and the type of environment of socialisation. From our analyses, we draw several implications related to TTI and the mitigation interventions implemented to contain the spread of SARS-CoV-2.

### More efficient, holistic approaches to TTI are needed

The emergence of a second and third wave of coronavirus cases in most European countries reveals how challenging it is to effectively implement the TTI strategy. In this paper, we have analysed how well Spain and Italy were able to test, trace and isolate their positive cases through the lens of an online, large-scale citizen survey.

The first pillar of the TTI strategy is *testing*. Several studies have emphasised the importance of minimising testing and tracing delays both in manual and digital contact tracing^11,14,15^. Kretzschmar et al.^15^ show that a testing delay of more than one day requires a tracing delay no longer than one day and a tracing coverage of a least 80% of contacts to keep the effective reproduction number below 1. A testing delay of 3 days or longer would make it impossible for the most efficient tracing strategies to reach a reproduction number below 1. The authors conclude that reducing the testing delays—by shortening the time elapsed between symptom onset and a positive test result—would be the most crucial factor to increase the effectiveness of contact tracing, assuming the immediate isolation of all positive cases, which, as we show next, is not the case in practical terms. According to our data, 41% of participants had to wait more than three days to receive their test results, while only 39% (Italian) and 37% (Spanish) of respondents had the test results within one day. Opposition to get tested has been found to be a potentially significant barrier in the TTI strategy^36^. Although our data might be biased towards people willing to collaborate, we have not found any evidence of a large percentage of the population in Italy and Spain refusing to get tested (3% in Italy and Spain).

In terms of tracing, while 75% of the respondents who reported having tested positive when they filled out the survey also reported having been called by a contact tracer, only 29% of the close contacts of a positive coronavirus case were traced. Moreover, we find that the types of contact that were mostly traced are different depending on the country. For example, Spain seemed to focus on tracing household members instead of tracing contacts from outside of the home. While tracing within the household is essential, the household members are likely to be already aware of their close contact with an infected individual in their own household. Moreover, previous work has reported that tracing only the household members has a limited impact on keeping under control the transmission in workplace and community settings^37–39^.

Hellewell et al.^11^ investigated the success of contact tracing depending on the initial number of infections, the reproduction number, and the amount of pre-symptomatic or asymptomatic transmission. Their results show that most scenarios with a reproduction number of 1.5 were controllable, with roughly 50% of contacts successfully traced. However, scenarios with a reproduction number of 2.5 required tracing more than 70% of the close contacts. Our results show that the tracing coverage in Spain and Italy was far from these figures. Moreover, the two countries were especially weak in tracing people with the highest number of close contacts (50+). Our findings also suggest that as the COVID-19 incidence increased, the contact-tracing capacity decreased, as also observed by previous literature^40^.

Given the limitations of manual contact tracing, various countries deployed digital contact tracing tools via a mobile app to complement the manual contact tracing efforts. Kretzschmar et al.^15^ claim that digital contact tracing on its own would be more effective than conventional manual contact tracing alone even with only 20% of app adoption, due to its inherent speed. In the best-case scenario, digital contact tracing alone could reduce the reproduction number by 17%. Several studies have shown the potential effectiveness of digital contact tracing using real-world contact patterns^39,41^ and in pilot studies in Switzerland, the United Kingdom (the Isle of Wight), and Spain (Gomera island)^42–44^. However, our data reveal the very limited role played by contact tracing apps. Despite having large adoption figures in our sample (31% in Italy and 19% in Spain), only 1% of respondents who reported having had a close contact with an infected individual that was revealed to them it via the app. In addition, a small fraction of those got tested. This limited role played by the digital contact tracing apps is the result of a conjunction of factors, including technological limitations, low levels of integration with local health systems, and delayed notifications. Thus, more detailed analyses on the real-world epidemiological effectiveness of the digital contact tracing apps would be needed^45^.

Finally, we observe discrepancies between officially reported and our survey data, particularly in Italy. According to officially reported data in Italy, more than 86% of the positive cases would have been contact traced after November 6th 2020 (see SI Fig. S20). According to our data, 75% of the close contacts of infected individuals were called, but only 61% of infected participants reported being aware that their close contacts had been called by contact tracers (see SI Fig. S11). Although this difference might arise from a different definition of close contacts, citizen surveys represent an alternative source of data to be compared with officially reported figures.

The final pillar in the TTI strategy is the effective *isolation* of all positive cases. According to our data, $54\%$ of participants aged 18-59 years old would be unable to self-isolate if needed. While most respondents were unable to self-isolate due to sharing their home with others, we identified additional age and gender-dependent reasons: psychological reasons are the most frequent amongst the youth (20% and 16% of participants aged 18-29 years old in Italy and Spain, respectively); and having to take care of children is most likely reported amongst women aged 30 to 59 years old (28% of women vs 13% of men within the same age group in both countries). Previous work has highlighted the importance of providing isolation infrastructure as a critical coronavirus pandemic control measure^16^. While Asian countries have provided hospitals and other facilities to support the isolation of all confirmed cases, European countries have only partly implemented such measures, mainly asking people to self-isolate in their homes, irrespective of their ability to do so^16,46^. Planning the necessary infrastructure to support isolation, particularly to the youth and families with young children is of paramount importance^47^.

### The importance of monitoring behaviour and perceptions

Our citizen survey allows us to compare the respondents’ self-reported behaviour before and after the pandemic containment strategies were deployed in Italy and Spain in the fall of 2020. Thus, through the lens of the survey, we can measure the impact of the government interventions on people’s behaviour, assess the compliance of the recommended individual protection measures, detect changes in behaviour that could lead to potential new waves of infections and estimate the population’s support to the government measures.

First of all, we find a decrease in the estimated number of distinct close contacts from outside the home and an increase of teleworking and unemployed respondents after several containment measures (e.g. restrictions or closures of point of interests such as museums, bars and restaurants; remote teaching activities; curfew) were implemented in Italy at the end of October 2020. This result provides supporting evidence to the effectiveness of these measures in limiting the number of close contacts among people and their potential in mitigating the spread of SARS-CoV-2. Coherent results were found in previous single-country^6^ or multi-countries^5,7,48^ data-driven studies as well as in previous modelling studies using fine-grained mobility data^49,50^. We also observed that a more limited impact of the mitigation interventions was found in Spain than in Italy, possibly due to the de-centralised approach to implementing these containment strategies in each of the 17 Autonomous Regions.

In Italy, we find that students and unemployed respondents reduced their close contacts the most, reaching a 58% reduction in the youngest demographic group (18 to 20 years old). This result might contribute to explain both the psychological difficulties reported by the youth in terms of their ability to self-isolate and the reported negative psychological impact (e.g. anxiety, depressive symptoms) of the pandemic on students^51,52^.

Regarding the adoption of individual protection measures, hand washing and face mask-wearing were the most widely adopted measures by Italian and Spanish respondents. Conversely, physical distancing and ventilation of indoor spaces were the least adopted measures. Even though roughly 82% of our respondents reported wearing a mask as much as possible, the context of mask wearing is important: masks might not be worn in the highest risk situations (e.g. private indoor spaces, with friends) and might be worn in lower risk, public environments, such as outdoors and streets. In fact, we find a noteworthy increase in the percentage of respondents who reported socialising in high-risk environments (i.e. indoors without protection measures) during the Christmas holidays, which might have been a side-effect of the closures of public gathering spaces, restaurants and bars during that time. This increased socialisation in high-risk environments might explain the rise of the third wave of coronavirus infections in Spain after the Christmas holiday period. We also found a misalignment between the perception of infection risk and real risk on some type of activities and places. For example, our data suggest that people consider beaches to be less safe than hairdresser salons and small stores, while indoor spaces would in principle be riskier than outdoor spaces, especially when social distancing measures are adopted^38,53,54^.

This misalignment in the perception of coronavirus infection risk seem to be a correlation between the perception of risk of a certain place/activity and how likely participants are to engage in such activities or places. For example, young participants (aged 18–29 years old) considered practising sports less risky than older participants (aged 60+ years old).

Based on these results, it would be essential to deploy public health communication campaigns to inform citizens about the actual infection risk of different activities and environments.

## Conclusion

During a pandemic, governments and societies must gauge real-time information about the effectiveness of non-pharmaceutical interventions, their impact on people’s behaviour and the population’s perceptions about them. However, deploying conventional surveys (e.g. pencil and paper surveys) during a pandemic has logistical and temporal challenges. Instead, online surveys allow a fast and cheap collection of data that might complement existing data sources and corroborate officially reported data.

The COVID19ImpactSurvey used in this paper was launched in March 2020 and has been regularly used by the Valencian Government^55^ to support and evaluate their decision making. With over 600,000 answers, it is one of the largest online citizen surveys related to coronavirus. The answers to the survey have enabled the Valencian Government to understand better people’s behaviour, perception, and compliance with the confinement measures; the economic, labour and psychological impact of the pandemic; their ability to self-isolate and the efficacy of contact tracing. Similar efforts have been made to collect symptoms information^56–59^ and behavioural data during the first wave^25,60–62^ of COVID-19. However, to the best of our knowledge, we are the first to collect such an extensive amount of data on self-reported human behaviour, and the control and mitigation strategies before and during the second wave of coronavirus infections in two countries.

Collecting a large sample of survey data (138,644 answers in two countries in our study) does not come without limitations. Citizen (online) surveys are particularly valuable tools in situations of data scarcity where informed and timely decisions are needed. Online surveys also allow monitoring people perception and behaviour, enabling the design of more effective non-pharmaceutical interventions and better education and communication with the public. However, even if the data size is large and confidence intervals are small, errors might compound and under- or over-estimate the results^63^. Moreover, even if we weighted^64^ the data in the attempt of mitigating the survey’s biases toward gender and age in each NUTS-3 statistical region^26^, and we deployed gender-balanced Facebook advertisement campaigns, the data is non-random and substantial biases are still present. For example, there are self-selection and sampling biases^65^ as the survey is filled out by volunteers who have learned about the survey via social media, WhatsApp, newspapers’ articles or Facebook ads, and who need to have access to a computing device (smartphone, tablet, PC) with an Internet connection. It is also possible that the survey is biased towards those people who are more likely to fill-in surveys, such as young and highly educated people. Then, respondents must be adults (at least 18 years old). Hence, students are only partially covered. Moreover, the survey does not collect any information about rurality, race, ethnicity and socio-economic classes, which might be important to assess the access to testing, tracing and the ability to isolate. We acknowledge all these biases challenge the estimation of inferential statistics, and might lead to overconfidence in incorrect inferences due to the collected large data sample and small confidence intervals^63^.

We also acknowledge that there might be a recall bias as respondents were asked about their behaviour, perceptions and situation in the last 7—sometimes 14—days (e.g. the number of close contacts in the last week). Finally, we analysed contact tracing via question Q11, which was not originally designed for this scope. However, it provides a valuable proxy of the number of close contacts and tracing efficacy, enabling useful comparisons between the two countries.

The collected survey allowed us to analyse the TTI and mitigation strategies in Spain and Italy, highlighting the limitations in such strategies and describing people’s behaviour during the COVID-19 pandemic. We anticipate that these results might stimulate a more thorough discussion about the implementation of TTI strategies during COVID-19 and future epidemics. We plan to expand the survey to other regions, focusing on developing economies in Latin America.

## Methodology

### Data collection and processing

We analyse a subset of the answers to the COVID19ImpactSurvey, an extensive, anonymous, online citizen survey about COVID-19^25^. Launched on March, 28th 2020, in Spain, the survey has since then collected over 600,000 anonymous answers from 11 countries, and it is available online at https://covid19impactsurvey.org/. Participants must declare being 18 years or older to be able to fill the survey. All research was performed in accordance with relevant guidelines/regulations. Informed consent was obtained from all users and the data were collected in de-identified form.

The project has been approved by the Responsible Research Board at the University Miguel Hernandez. The Board is composed of the following members: Alberto Pastor, Tomas de Domingo, Javier Saez, Eugenio Vilanova, Vicente Micol, Antonio Guerrero and Ana Maria Madariaga. The Responsible Research Board at the University Miguel Hernandez has the following charter: (1) guarantee that the research is performed in an appropriate legal, ethical and safe framework; (2) improve the quality of the research^66^.

We analysed the answers of 24 questions in the survey which collect information about the participants’ demographic and household situation (Q1–Q6); their social contact behaviour (Q9–Q11); their support for the government measures deployed to contain the spread of COVID-19 (Q12) and for a potential lockdown (Q13); the economic impact of the pandemic in their lives (Q17–Q18); their ability to self-isolate if needed (Q19); the individual protection measures that they adopt to protect themselves against COVID-19 infections (Q20); whether they have been tested (Q24) for COVID-19; their willingness to get tested for COVID-19 infections (Q25) and the psychological impact of the pandemic in their households (Q26). There are gaps in the numbering, as we removed purely lockdown related questions from the original survey that were no longer relevant to participants in order to make room for questions around TTI. We describe in SI Table S1 all the questions.

We analyse the data from Spain and Italy for the time period between June 2020 and December 2020. SI Table S3 and Figure S4 summarise the statistics of the data.

Survey answers were collected from volunteer respondents who learned about the survey via social media channels, word-of-mouth, universities and news organisations. We used Facebook and Instagram ads as an additional channel to recruit volunteers. This approach gave us a straightforward method to obtain a broad sample of users across each country. We used the same ads, images and budgets in both countries. We did not use any targeting feature except for gender, where we used separate budgets to balance the numbers of male and female respondents. The cost-per-successful-response was €0.24 and €0.11 for men and women respectively in Spain, and €0.13 and €0.07 for men and women respectively in Italy. Most of the cost difference between men and women could be explained by their higher willingness to click on ads, where women clicked on 2.1% of ads shown vs 1.3% of men. Both men and women had similar completion rates (54%) once they began answering the survey.

The age and gender distribution of our sample might be different from the total population distribution. Thus, we follow the methodology described in Oliver et al.^25^ and re-weight the answers such that the resulting gender and age distributions match the official statistics of each NUTS-3^26^ statistical region in Spain (i.e., provinces, islands and the autonomous cities of Ceuta and Melilla) and Italy (i.e., provinces). We compute the weights following the official data reported by the Spanish National Institute of Statistics (INE) and the Italian National Institute of Statistics (ISTAT) of 2020.

To further validate our methodology, in April 2020 we commissioned an IPSOS.digital FastFacts panel of a cohort of 1000 representative general population respondents aged 18–65 in Spain. This validation was done after we started using Facebook ads, but before the time period covered by this paper. The results of the IPSOS panel were generally within the margin of error of our survey results for the same time period and ages.

### Selection of the time periods

We divided our analysis into two phases: (1) Phase I—new normality and (2) Phase II—second wave. In Italy, the Phase I period starts on June 15th, 2020 when there was a relaxing of many of the implemented confinement measures, including cinemas, theatres and discos^67^. However, we started our survey in Italy on July 31st. Phase I ends on October, 26th 2020 with the deployment of the first mitigation measures to reduce the spread of COVID-19^68^ and the subsequent national colour system imposed to restrict movements and social gatherings^33^. In Spain, Phase I starts on June, 21st 2020 with the “new normality” phase, when most of the confinement measures were lifted and minor restrictions, such as defining a maximum occupancy in shops are handled by each autonomous community independently^69^. It ends on October, 25th 2020 with the re-deployment of a state of emergency across the country^34^.

### Confidence intervals

Throughout the paper we report Confidence Intervals (CI) to ease the comparison between percentages of different groups or states, especially when percentages are very similar. To do so, we interpret our percentages as a Binomial outcome of a series of success-failure experiments (Bernoulli trials). Then, we compute the 95% CI approximating the distribution of error about a binomially-distributed observation, ${\hat{p}}$, with a normal distribution^70^. More formally:

$$\begin{aligned} {{\hat{p}}} \pm 1.96 \sqrt{\frac{{{\hat{p}}} \left( 1 - {{\hat{p}}}\right) }{n}} \end{aligned}$$

where ${\hat{p}}$ is the percentage of positive outcomes, measured with *n* trials.

### Welch’s t-test

We used the Welch’s t-test^71^ to test whether two populations have equal means. The test is based on the Student’s t-test^71^ and computes the t statistics as: $t = {\overline{X}}_1 - {\overline{X}}_2 / \sqrt{ {s_{{\bar{X}}_1}^2} + {s_{{\bar{X}}_2}^2} }$ where ${\overline{X}}_{i}$ and $s_{{{\bar{X}}}_{i}}$ are sample mean and its standard error, respectively. The degrees of freedom $\nu$ are instead computed as $\nu \approx {{\left( \; {s_1^2 \over N_1} \; + \; {s_2^2 \over N_2} \; \right) ^2 } / { {s_1^4 \over N_1^2 \nu _1} \; + \; {s_2^4 \over N_2^2 \nu _2 } }}$.

### LOESS regression

To fit the testing capacity trend, we used a standard LOESS method released by scikit-misc^72^ to estimate the main trend and its confidence intervals. However, this method might be influenced by outliers. Thus, we in the SI we added a comparison between the non-robust and robust LOESS estimators^30^. We find a significative correlation (Spearman’s r 0.94, p-value<0.0001) between the trend of the two methods, suggesting that outliers do not strongly influence our interpretation of the results.

## Supplementary Information

Supplementary Information.

## Data Availability

The data that support the findings of this study are available on request from the corresponding author M.D.N.
