# Supplementary material for: The impact of control and mitigation strategies during the second wave of coronavirus infections in Spain and Italy
Source: Sci Rep. 2022 Jan 20;12:1073. doi: 10.1038/s41598-022-05041-0 (PMC8776768; doi:10.1038/s41598-022-05041-0)
Supplement: Supplementary file 1 — Supplementary Information. [file 41598_2022_5041_MOESM1_ESM.pdf]

The Impact of Control and Mitigation Strategies during the Second Wave of Coronavirus Infections in Spain and Italy
Supplementary Information

Marco De Nadai,[1] Kristof Roomp,[2] Bruno Lepri,[1] and Nuria Oliver[3]

[1]*Fondazione Bruno Kessler (FBK), Italy*

[2]*Microsoft - United States, USA*

[3]*ELLIS Unit Alicante Foundation, Spain*

CONTENTS

S1. Survey questions 2

Community engagement 6

Perception of the Government Measures 8

S2. Description of the Italian restrictions 10

S3. Description of the Spanish restrictions 11

S4. LOESS Robustness 11

S5. Additional figures 12

S5.1. Perception of COVID-19 risk in places and activities 19

S5.2. Trends of weekly close contacts 27

S6. Additional tables 27

References 33

S1. SURVEY QUESTIONS

Table S1 depicts the questions whose answers have been analysed in this paper. The survey has a total of 24 questions. However, there are a few conditional questions such that the number of the questions is that a person might answer could be less than 24. There are gaps in the numbering since during the lifetime of the survey questions regarding the lockdown behaviour were removed as they were no longer relevant.

**Question**	**Possible answers**
**Demographic and Household information**	
Q1. What is your age range?	[18-20; 21-29; 30-39; 40-49; 50-59; 60-69; 70-79; 80+]
Q2. What is your gender?	[Male; female; another gender]
Q3. Postal code/Zip code	Text entry
Q4. Type of home	[Single family house; apartment/flat; shared apartment/flat; other shared accommodation; other]
Q5. Number of people in the home (including you)	[1; 2; 3; 4; 5+]
Q6. Age(s) of people in the home (excluding you, check all that apply)	[10 or less; 11-20; 21-29; 30-39; 40-49; 50-59; 60-69; 70-79; 80+]
**Tracing**	
Q9. In the last seven days, approximately how many different people that live outside your home have you had close contact with? (meaning more than 15 minutes and a distance closer than 2 meters)	[No one; 1-2; 3-4; 5-9; 10-19; 20-29; 50+]
Q9_1. (only for those who had close contact with someone from outside the home) What was your most frequent type of close contact in the last seven days from outside your home? [We added this question on October 10, 2020]	[coworker; client; family/friends; school/university; concert/show/disco/club/conference; shopping; as a customer (bank, office); doctor/clinic/hospital; travel (including public transportation)]

Q10. In the last seven days, what was the most common location that you spend time socializing with your friends, relatives and acquaintances that live outside your home? [We added this question on November 12, 2020]	[Private house, apartment, residence or club while being strict about masks, distancing and ventilation; Private house, apartment, residence or club while more relaxed about masks, distancing and ventilation; Restaurant/coffee shop/bar/disco (indoors); Restaurant/coffee shop/bar/disco (outdoors); workplace; School/university; on the street, park, other public space; At the beach; in nature outside the city; other location; I did not socialize with anyone that lives outside my household]
Q11. Have you had physical contact with someone diagnosed with coronavirus (in the last seven days)? (check all that apply)	[Member of household; Family outside household; friend or acquaintance; coworker; Cleaning staff/caretaker/etc; Patient (in case of medical staff); client/customer; student; Unknown person (I was notified by the app or a contact tracer); None that I know of]
Q11_1. (only for those who respond that they had had a close contact with an infected individual) Have you been contacted by a doctor or a contact tracer about your close contact with someone infected with coronavirus? [We added this question on August 10, 2020]	[Yes; No; No, but I was notified by the app]
Q24_3. (only for those who tested positive) Did your doctor or health authority ask you to identify your close contacts in order to trace the infection? [We added this question on July 24, 2020]	[No, I was not asked about my close contacts; Yes, but none of my close contacts were called (to my knowledge); Yes, and some of my close contacts were called; Yes, and some of my close contacts were called and tested for coronavirus]
**Testing**	
Q24. Have you been tested for coronavirus?	[a) Yes, I am waiting for my result; b) Yes, the test said that I have coronavirus; c) Yes, the test said that I had coronavirus, but I am now recovered; d) Yes, the test said I do not have coronavirus (recently); e) Yes, the test said I did not have coronavirus (more than one month ago); f) No; I prefer not to answer]

Q24_1.(only for those who respond having been tested) In total, how long did it take (or are still waiting) to get an appointment, get tested for coronavirus and receive the results? [We added this question on September 25, 2020]	[1 day; 2 days; 3-4 days; 5-7 days; 8-13 days]
Q25. Are you currently trying to get tested for coronavirus due to having symptoms or having been recently exposed to an infected individual?	[Yes, I have an appointment to get tested and I am waiting for it; Yes, I am trying to get an appointment to get tested; Yes, but there are no tests available; Yes, but I do not know how to get tested; Yes, but the test is too expensive; No, but I want to take it; No, I do not think I need it; No, and I would refuse to take it; I prefer not to answer]
**Isolating**	
Q19. If you were diagnosed with coronavirus and had to be quarantined for at least 2 weeks, would you be in any of the following situations? (check all that apply)	[I could not isolate myself from other people in my home; I would have to continue taking care of other people (children, parents...); I depend on a caregiver; It would be difficult for me to get medical leave from work; I could lose my job; I could not afford it financially; It would be impossible for me psychologically; I would be afraid of discrimination or stigmatization; None of the above]
**Behavior and Perception**	
Q12. Do you think there should be a another lockdown if there is another wave of coronavirus?	[Yes, similar to the first lockdown; Yes, but stricter than the first lockdown; Yes, but less strict than the first lockdown; Yes, but only for people who are at risk; No, the economic and/or social cost would be too high; No, it would not be accepted by the population; I do not think there will be another wave; None of the above]
Q13. Do you believe that the measures the government has taken are enough to contain the spread of coronavirus?	[No, but should be stricter; Yes, are about right; Yes, but are too strict; Prefer not to respond; I do not know]

Q15. Which of the following activities do you think can be done with a low risk of coronavirus infection? (check all that apply)	[Practicing individual sports; Having friends visit you at home; Attending religious services with limited seating; Attending school like in some European countries; Going to small businesses with appointment (hairdresser, etc); Going to small shops while maintaining a safe distance; Having drinks at a bar on an open terrace with a group of people; Going to restaurants with limited seating; Receiving treatment at a hospital; Taking public transportation with space between seating; Going to the beach; Traveling by air; None of the above]
Q20. Do you take any of the following measures to prevent the transmission of the coronavirus? (check all that apply)	[I wear a mask as much as possible; I avoid crowded situations; I do not shake hands, give hugs or kisses to anyone who live outside my home; I regularly disinfect/wash my hands; I keep my physical distance of at least 1.5 meters (6 feet) from others; I limit the number of people that I am in close contact with; When indoors, I make sure there is good ventilation; I have installed my government's contact tracing app on my phone; None of the above]
Q21. Do you take any of the following measures to prevent the transmission of the coronavirus? (check all that apply)	[I wear a mask as much as possible; I avoid crowded situations; I don't shake hands, give hugs or kisses to anyone who live outside my home; I regularly disinfect/wash my hands; I keep my physical distance of at least 1.5 meters (6 feet) from others; I limit the number of people that I am in close contact with When indoors; I make sure there is good ventilation; I have installed my government's contact tracing app on my phone; I would be willing to get vaccinated immediately when the coronavirus vaccine is available; None of the above]
**Tele-work**	
Q17. Have you worked any time since March 1st, 2020? (before the beginning of the coronavirus crisis)	[Yes; No; No, I am a student; No, I am retired]
Q17_1 (only for those who respond that they have worked) Have you gone to work in the last seven days?	[Yes; Yes, with reduced hours; I am on leave or teleworking because I am in quarantine due to coronavirus; No, but I am teleworking; No, I am on paid leave (vacation, maternity, etc); No, I am on unpaid leave; No, I have lost my job or stopped working]

Q18. What is your main job?	[Essential services (police, firefighter, medical personnel); Retail; Manufacturing; Health and social services; Hospitality (restaurants, bars, hotels, etc); Education; Government or defense; Construction; Transport; Administrative assistant and similar; Professional, technical or scientific services; Farming, fishing or other food production; Press or communication; Household employee; Financial; Artist, recreation and entertainment; Sanitation, cleaning, garbage collection; Other services]

TABLE S1: COVID19ImpactSurvey questions analyzed in this study.

Community engagement

The cooperation of the population by complying with social distancing and personal protection measures is essential to control the transmission of coronavirus and maintain a low reproduction number [1]. Thus, we analyse the participants' perception of the risk of getting a coronavirus infection associated with performing different activities and/or going to various places (Q15 in SI Table S1). We also analyse the personal protection measures they report adopting to prevent a SARS-CoV-2 infection (Q20 in SI Table S1) and whether they telework regularly (Q17, Q17_1 and Q18 in SI Table S1).

We find some differences in the behaviour of participants in the two countries. Hence, we report the results for each country separately. Figure S1A suggests that Spanish respondents think that fewer places are safe when compared to what Italians think. For example, only 31% of Spaniards consider restaurants to be places with a low risk of getting COVID-19 compared to 50% of Italian respondents. Similarly, schools are perceived safer by Italian (39%) than by Spanish (27%) participants. Moreover, we identify large gender-based differences in the perception of risk. For example, men are more likely than women to consider that individual sports (Italy: 74% vs 65%, Spain: 79% vs 73%), restaurants (Italy: 54% vs 47%, Spain: 35% vs 29%) and having friends at home (Italy: 40% vs 33%, Spain: 27% vs 22%) can be performed with a low risk of getting a coronavirus infection. This perception of safety also changes with time: as the cumulative incidence of COVID-19 cases rises, we observe a consistent increase in the perception of risk of all activities and places in both countries. However, the ranking of the activities/places remains the same throughout the study: air travel is consistently considered the activity with the highest risk of getting infected with COVID-19 and practising individual sports the activity with the lowest risk

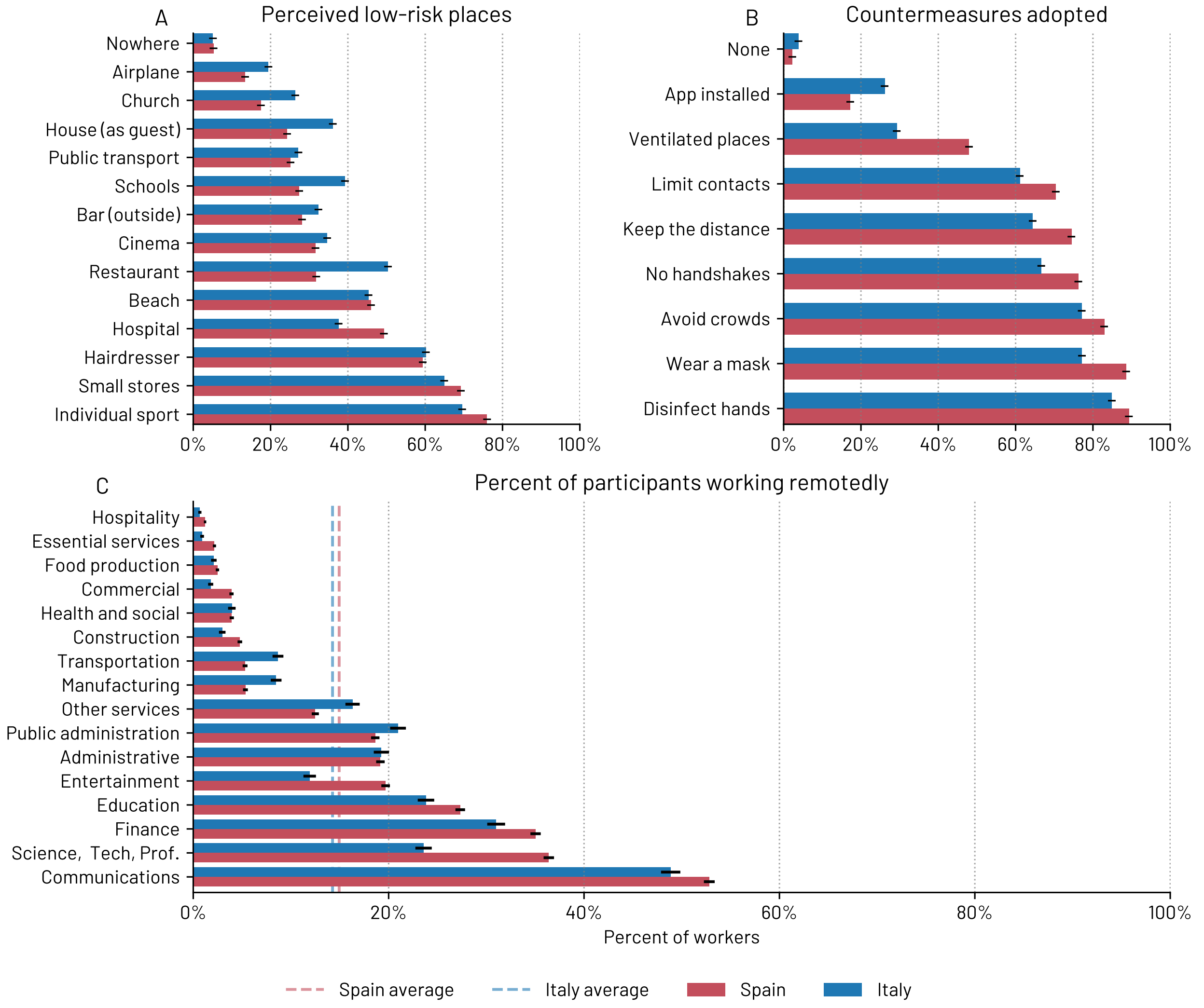

FIG. S1. Human behaviour in the Phase I - *new normality*. A) Perception of safety (in terms of risk of getting a COVID-19 infection) for different activities/places in Spain and Italy. B) Individual COVID-19 protection measures adopted in Spain and Italy. C) Percentage of workers that report teleworking per sector, in Spain and Italy. In this figure, we report the 95% confidence intervals.

of infection. We refer to the SI Figure S14 and Figure S15 for additional details.

Figure S1B shows the personal protection measures adopted to prevent a coronavirus infection. Again, we observe that Spanish respondents are more cautious about COVID-19 and adopt more countermeasures than Italians in all areas but the digital contact tracing app installation, where more Italian respondents report having the app installed than Spanish respondents (Italy: 26%, Spain: 17%). The most notable difference is found in the adoption of ventilation: 48% of Spanish vs 29% of Italian participants report ensuring good ventilation indoors.

We identify gender and age differences, with women and older respondents being generally more

compliant and cautious than men and the youth. For example, women are more likely than men to report avoid shaking hands (Italy: 72% vs 61%, Spain: 78% vs 74%), limit their contacts (Italy: 66% vs 56%, Spain: 75% vs 66%) and disinfect hands (Italy: 89% vs 80%, Spain: 92% vs 86%). We report more information in SI Figure S16 and Figure S17.

Finally, most countries deployed public communication campaigns and programs to promote telework and hence reduce the risk of infection at work. Figure S1C shows the percentage of participants who report remote working in the new normality phase in Spain and Italy. The categories that have more remote workers are Communications, Science/Tech/Professional and Finance, which are also amongst the most digital sectors [2]. Although the average is similar in both countries (around 15%) and considerably larger than the values before the COVID-19 pandemic (∼5% in Italy and ∼6% in Spain in 2019 according to a recent European report [3]), we observe differences per sector per country, particularly in the Communications (52% vs 48%), Science/Tech/Professional (38% vs 23%) and Entertainment (19% vs 11%) sectors. In these sectors, a larger percentage of Spanish respondents report teleworking when compared to Italian respondents, which might be a consequence of Spain having a larger Digital Technology Integration Index when compared to Italy (42 vs 33 according to [3]).

Spanish respondents and women in both countries tend to be more compliant in adopting personal protection measures –such as wearing facial masks– and more conservative in their estimation of COVID-19 risk associated with different activities/places. This identified gender difference is aligned with previous studies on attitudes and behaviours during the COVID-19 pandemic [4] as well as with the literature showing that women tend to be more risk-averse and more open to government interventions than men [5, 6]. Overall, these results highlight the challenges posed by behavioural changes in response to new risks and the need for targeted risk communication strategies [4].

Perception of the Government Measures

As the number of confirmed COVID-19 cases rose again after July 2020, governments were forced to balance a new implementation of mitigation policies that would combine swift reactions and softer confinement measures to slow down the growth in the number of positive cases while minimising their economic and social impact. Thus, public health experts and policymakers needed to persuade their citizens to support and comply with the implemented measures.

Figure S2A shows how public opinion related to the implemented COVID-19 measures evolved over time in Italy and Spain (Q13 in SI Table S1. In Italy, as soon as the number of confirmed

cases rose in the first week of October 2020, those who thought that the government should implement more measures increased from less than 40% to more than 50% around the first week of November. Consequently, the percentage of people thinking that the Italian policymakers had implemented enough measures fell to around 20%. Note that Italy implemented a systematic pandemic containment regulation between the end of October and the first week of November. Surprisingly, in Spain, the percentage of respondents demanding more measures decreased in October 2020, even if the number of confirmed COVID-19 cases increased. This phenomenon might be related to the so-called pandemic fatigue [7], which makes NPIs less effective. Interestingly, the percentage of respondents demanding more measures only increased *after* the peak of infections in the second wave was reached in November 2020.

We also observe that the public opinion about a possible second lockdown changed over time in Italy and Spain (Q12 in SI Table S1). Both in Italy and Spain, we find an increase in the percentage of participants asking for a second lockdown as soon as the second wave hit the two countries in October 2020 (see Figure S2B and Figure S2C). Notably, in Italy, the percentage of respondents asking for a second lockdown increased by around 20% from the first week of October to the first week of November 2020; in Spain, the increase was over 20% between the first week of September until the end of the year.

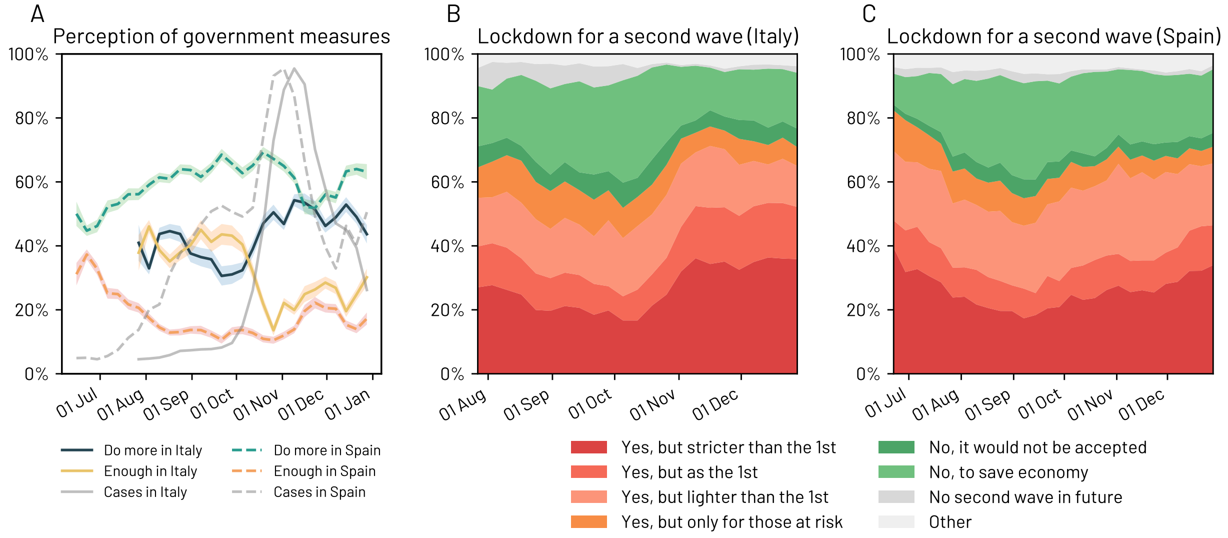

FIG. S2. Participants' perception of the Government measures and support for a potential lockdown change over time. In Italy (A-B) they seem influenced by the number of confirmed COVID-19 cases. We note that our analysis starts on July 31st and June 21st in Italy and Spain, respectively. In A) we report the 95% confidence intervals.

S2. DESCRIPTION OF THE ITALIAN RESTRICTIONS

With the rise of confirmed COVID-19 cases in Italy in early October, the Italian Prime Minister imposed on October 7th 2020, the compulsory use of face masks in public spaces [8]. Between the 13th and the 26th of October, stricter rules were implemented, such as in the opening hours and maximum allowed capacity of restaurants and bars [9–11]. Museums, theatres, cinemas, gyms and swimming pools were closed. One month after the rise of the confirmed number of COVID-19 cases, the government defined a three-tiered system on November 6th 2020 [12]. Each region was assigned a tier depending on several quantitative indicators: i) the level of transmission (e.g. the effective reproduction number, the number of positive cases in the past two weeks, the number of novel outbreaks), ii) the resilience and effectiveness of the control strategies (e.g. the rate of positive tests, the number of positive cases that were contact traced, the temporal delay between the symptoms onset and the COVID-19 test outcome), and iii) the burden on the healthcare system (the hospital occupancy rate for COVID-19 cases, the number of intensive care units). High schools were closed on November 6th, and students were taught remotely; universities were encouraged to teach remotely.

S3. DESCRIPTION OF THE SPANISH RESTRICTIONS

Spain did not have a centralised COVID-19 mitigation strategy after the central government lifted the state of alarm on June 21st 2020 [13]. Hence, during the time period between June 21st and October 25th 2020, each of the 17 autonomous regions made their own decisions related to the pandemic, imposing restrictions as needed. However, in October 2020, all the autonomous regions in Spain (except for Madrid and the Basque Country) decided to adopt a common system to determine the pandemic risk via a set of indicators and implemented the same mitigation measures to slow the spread of COVID-19. They agreed to adopt a 5-level risk system ranging between *new normality (no risk)* to *extreme risk* based on their healthcare occupancy levels, the cumulative incidence, the positivity rate and the percentage of traced cases. Moreover, on October 25th, 2020, the government declared again a state of alarm [14], which enabled establishing a national curfew and gave power to local authorities to ban travel across regions, provinces and municipalities if needed. In terms of the measures, while every Autonomous Region decided to adopt their own, they were similar across regions and included limiting the mobility in the evening and at night, implementing regional/local confinements, reducing the maximum number of people in social meetings to 4-10 individuals, limiting the maximum capacity in locales to 25-50% and partially or totally closing shops in non-essential sectors, restaurants and bars.

S4. LOESS ROBUSTNESS

In the main paper, we use the LOESS method to estimate the Italian trend of the capacity to trace positive people. The method we use is not robust to the effects of outliers (like other least squares methods) but allows to estimate the confidence intervals of the trend.

We here verify whether the iterative robust LOESS regression [15] has different outcomes. Figure S3 shows that the two methods produce very similar trends and have a Spearman's r correlation of 0.95 pvalue$<$0.0001. Thus, our regression is not greatly influenced by outliers.

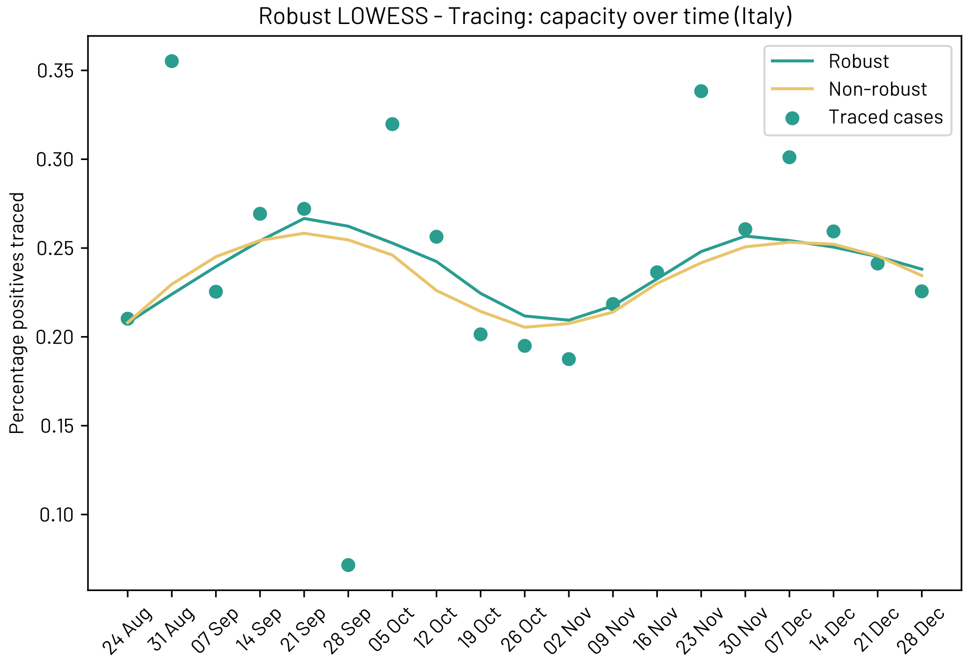

FIG. S3. Robust LOWESS fit on the tracing capacity data.

S5. ADDITIONAL FIGURES

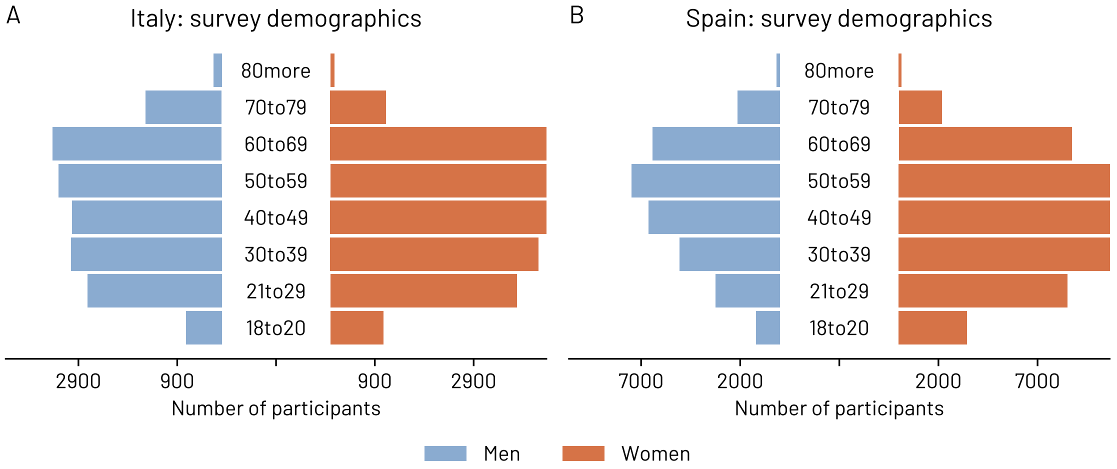

FIG. S4. Demographic composition of the respondents in Italy and Spain.

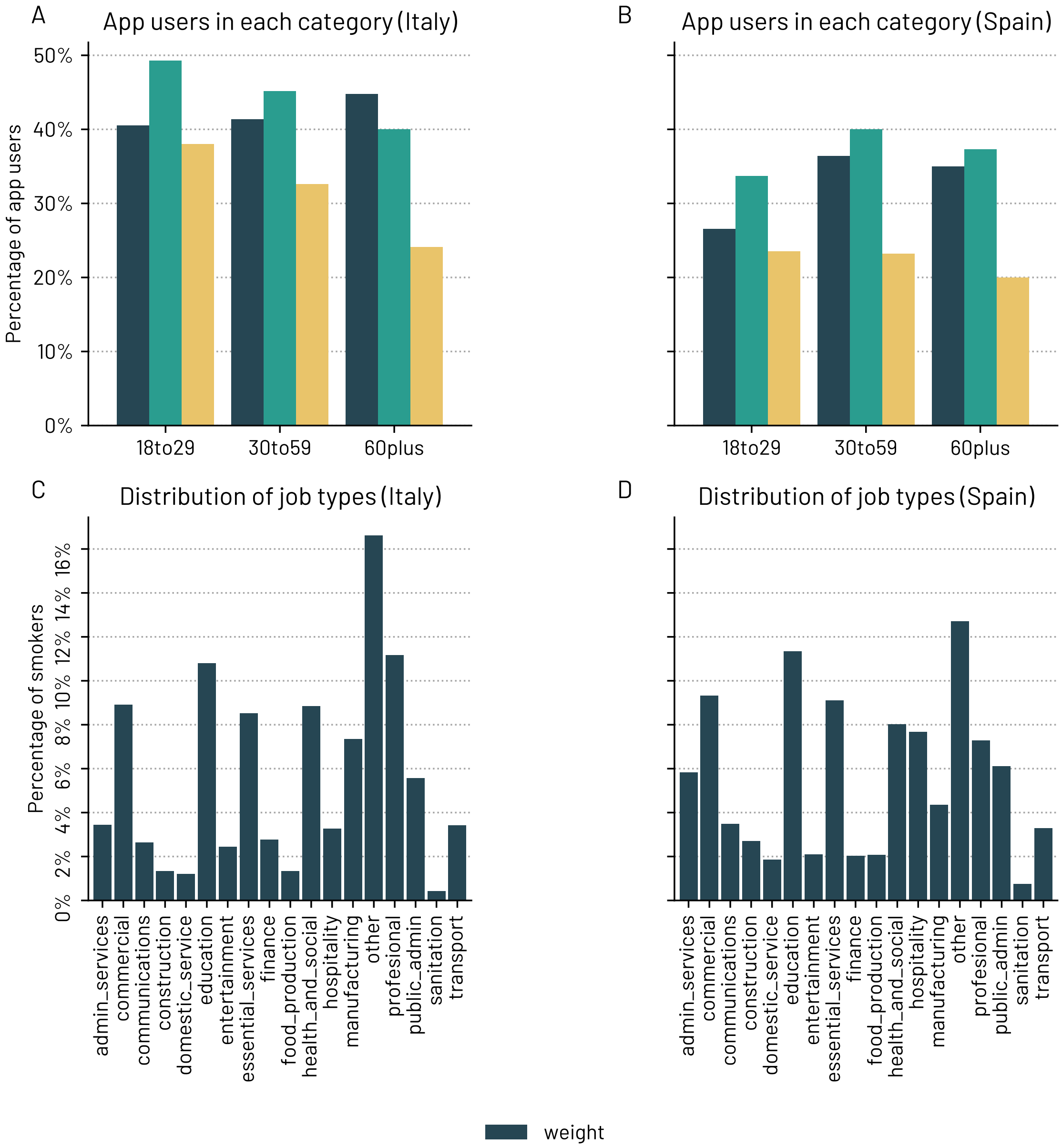

FIG. S5. Distribution of app users (A-B) and job types (C-D) in the respondents per age group in Italy and Spain.

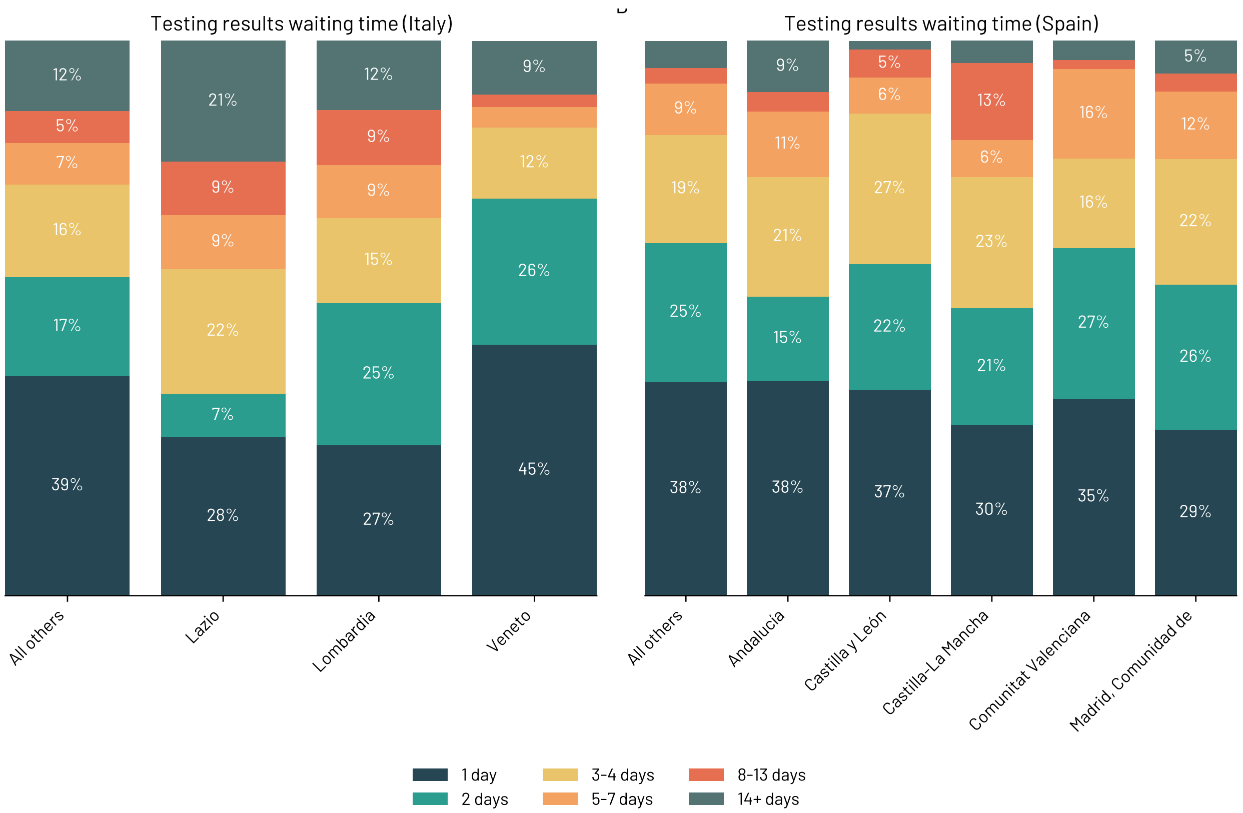

FIG. S6. Time to get the test results for each region in Italy (A) and Spain (B).

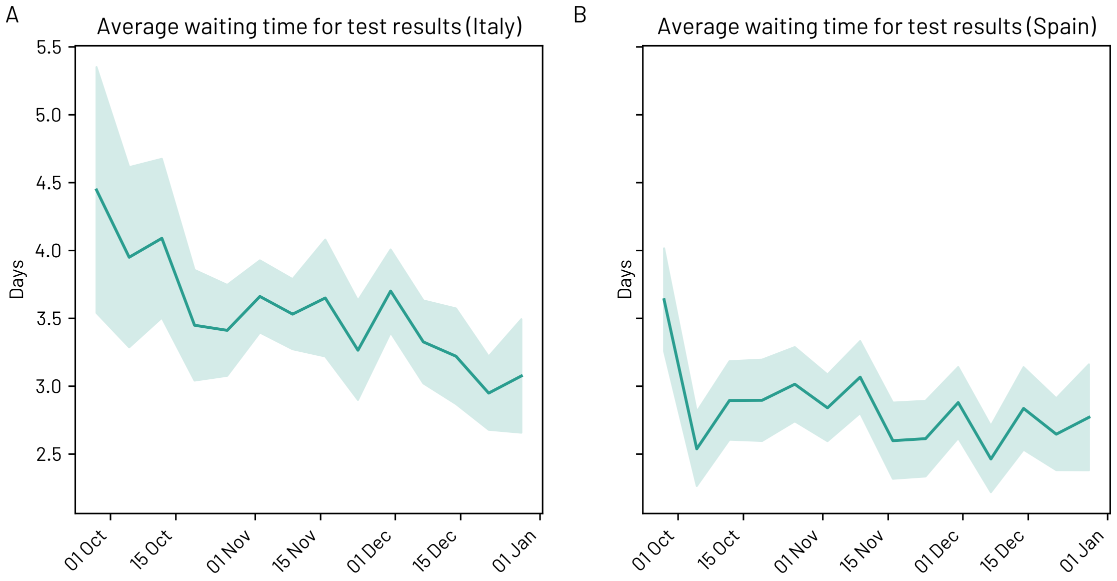

FIG. S7. Time to get a test results, over time, for Italy and Spain during the Phase I - *new normality*.

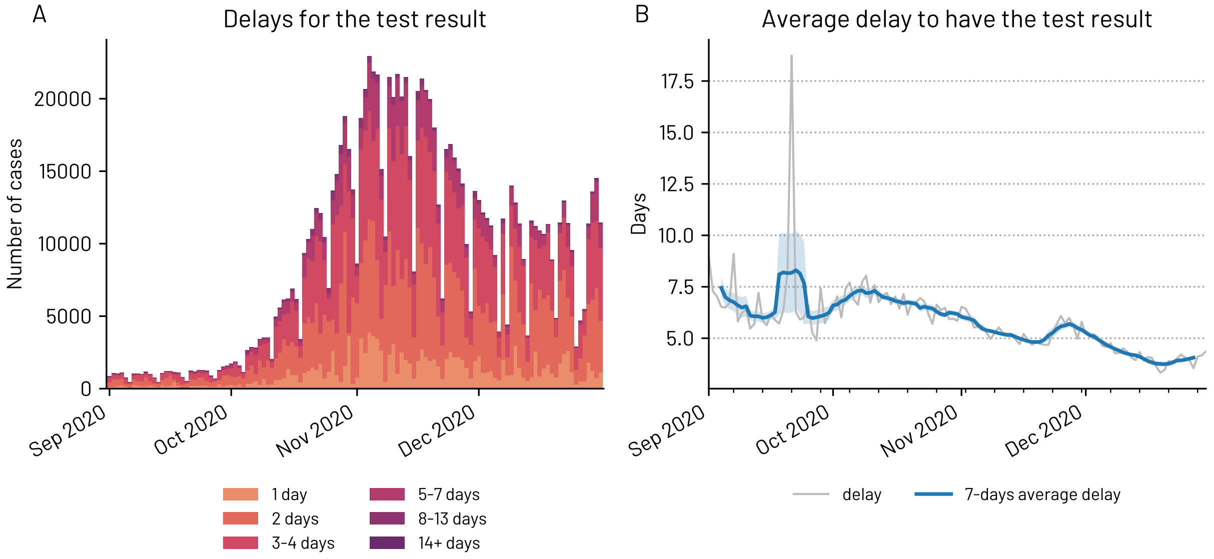

FIG. S8. Italian delays to get the COVID-19 result. Official data from the ISS. In B) we report the 95% confidence intervals.

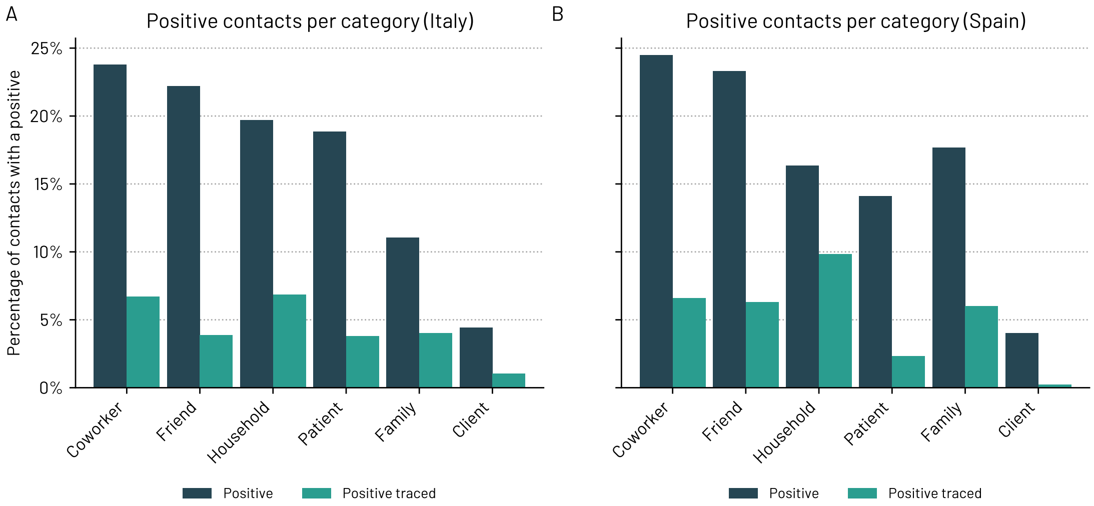

FIG. S9. A-B) The vast majority of respondents who reported having had a close contact with a positive case were not traced nor contacted during the Phase I - *new normality*. A person is traced if the doctor or the health authority identified his/her contacts. A person is contacted when some of the close contacts were called by the doctor or the health authority. C-D) Percentage of contacts with a positive and positive traced by contact type.

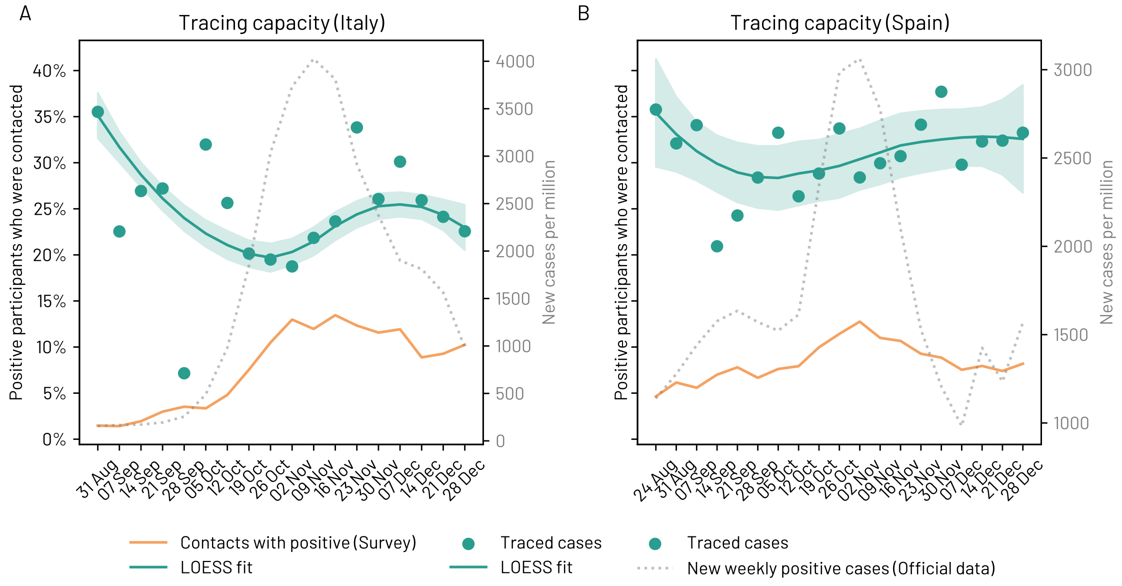

FIG. S10. Tracing capacity over time and daily number of COVID-19 positive cases. We report the 95% confidence intervals.

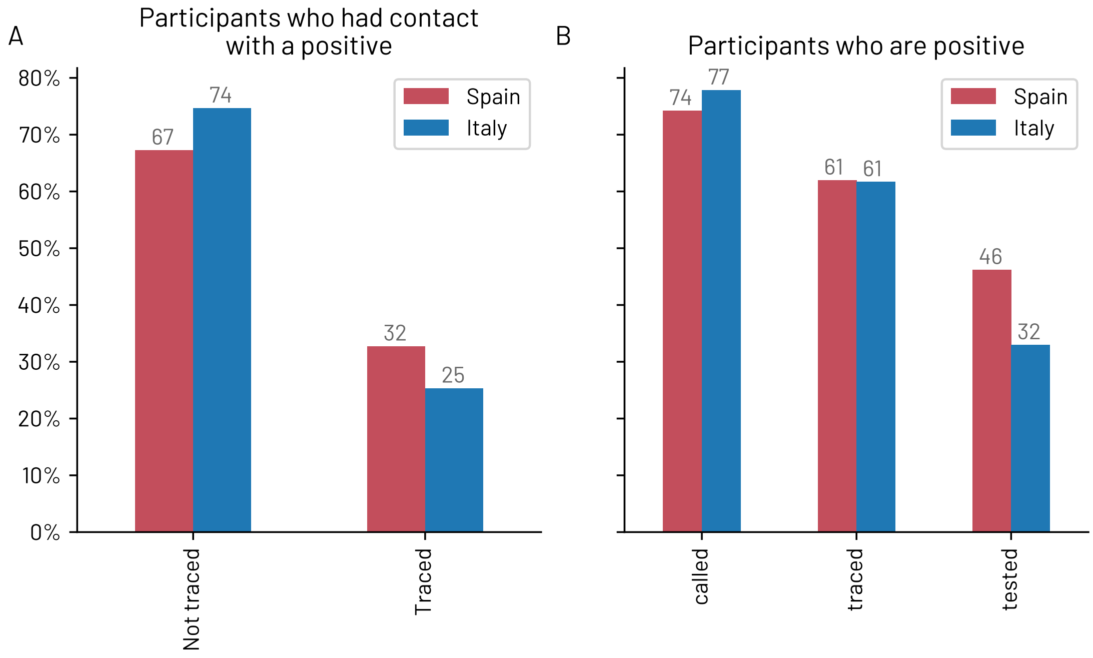

FIG. S11. Tracing statistics during the Phase I - *new normality*. A) Percentage of participants who reported having had a close contact with a positive case and having been contacted by the health authority. B) Percentage of respondents who reported testing positive when they filled the survey and having been contacted by the health authority.

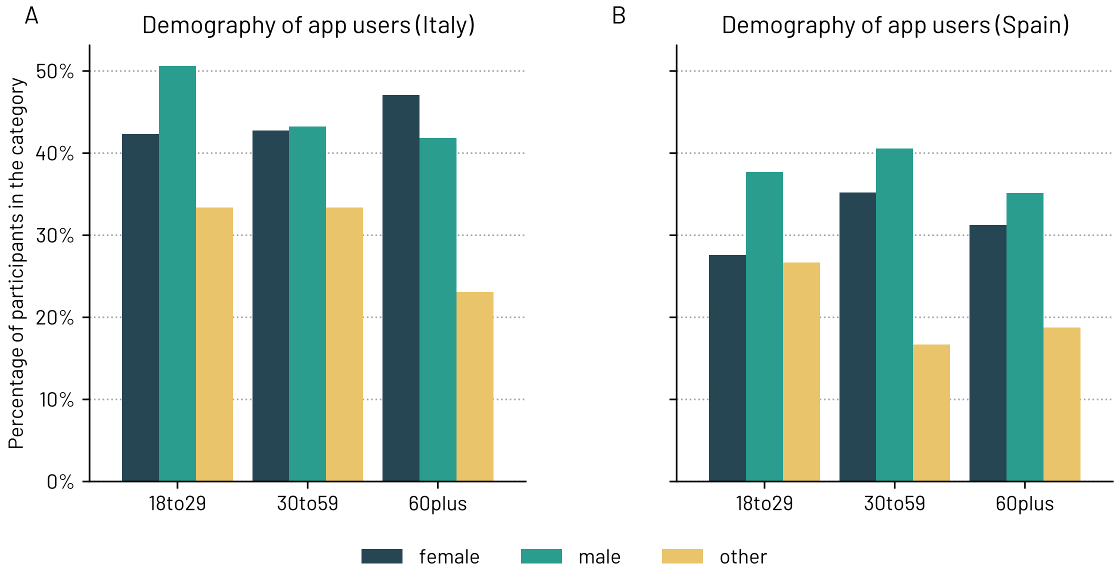

FIG. S12. Percentage of app users per each demographic category in A) Italy and B) Spain during the Phase I - *new normality*.

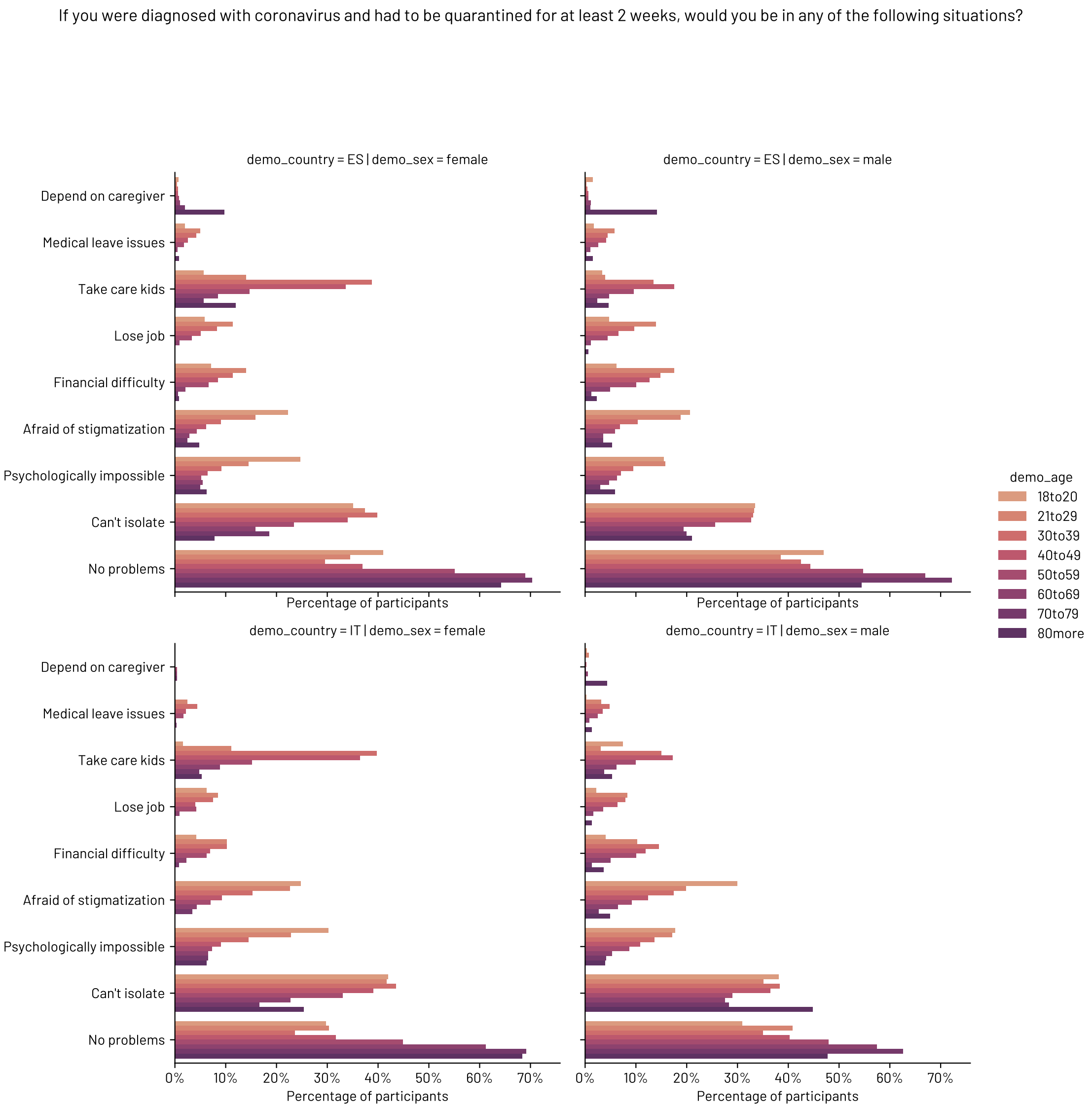

FIG. S13. Reported barriers in the case of being diagnosed with coronavirus and having to be quarantined for at least 2 weeks during the Phase I - *new normality*.

S5.1. Perception of COVID-19 risk in places and activities

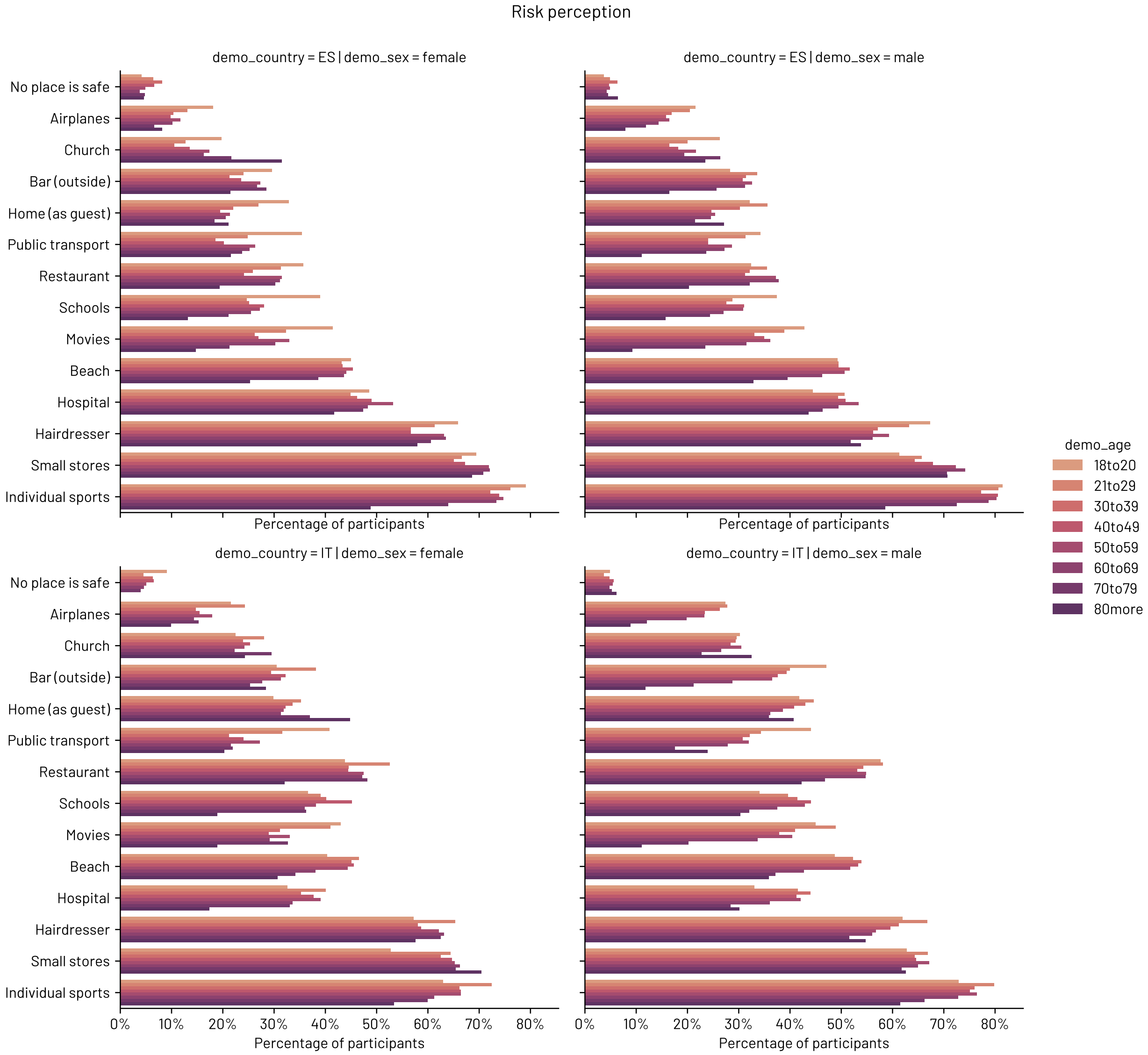

FIG. S14. Perception of safety per place, country, and demographic group during the Phase I - *new normality*.

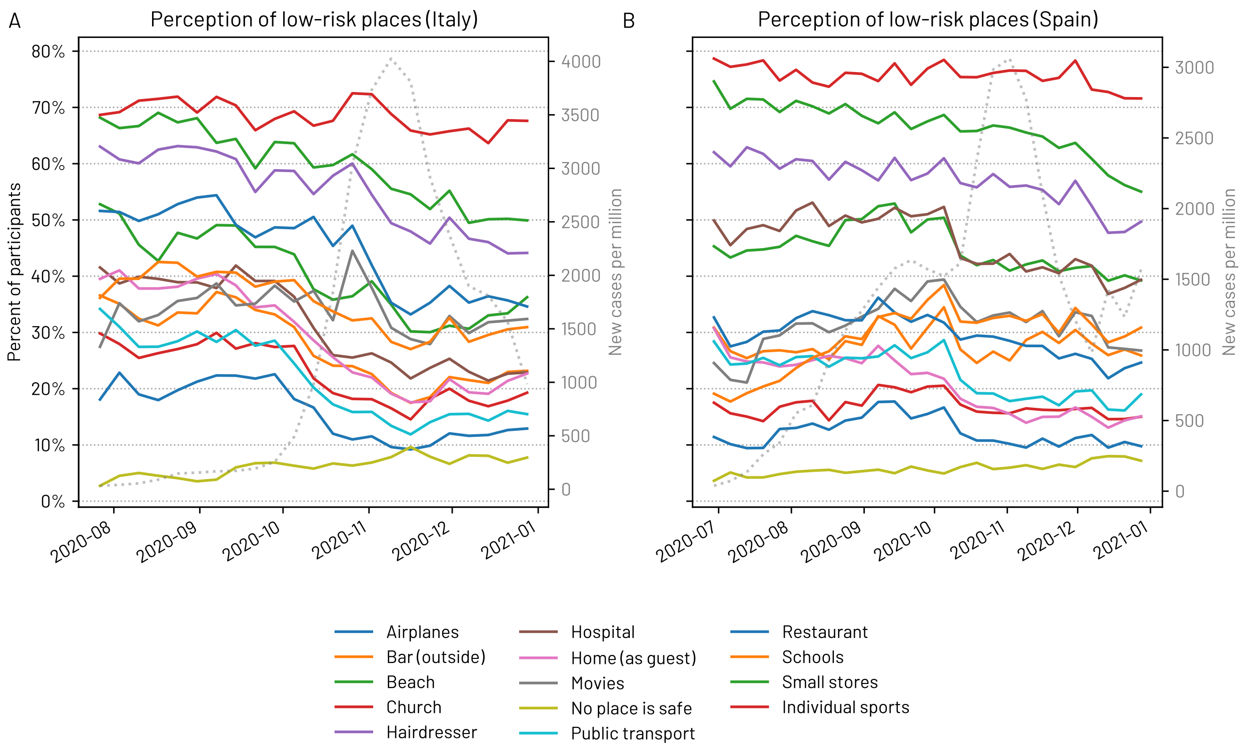

FIG. S15. Evolution of the perception of safety of activities and places over time.

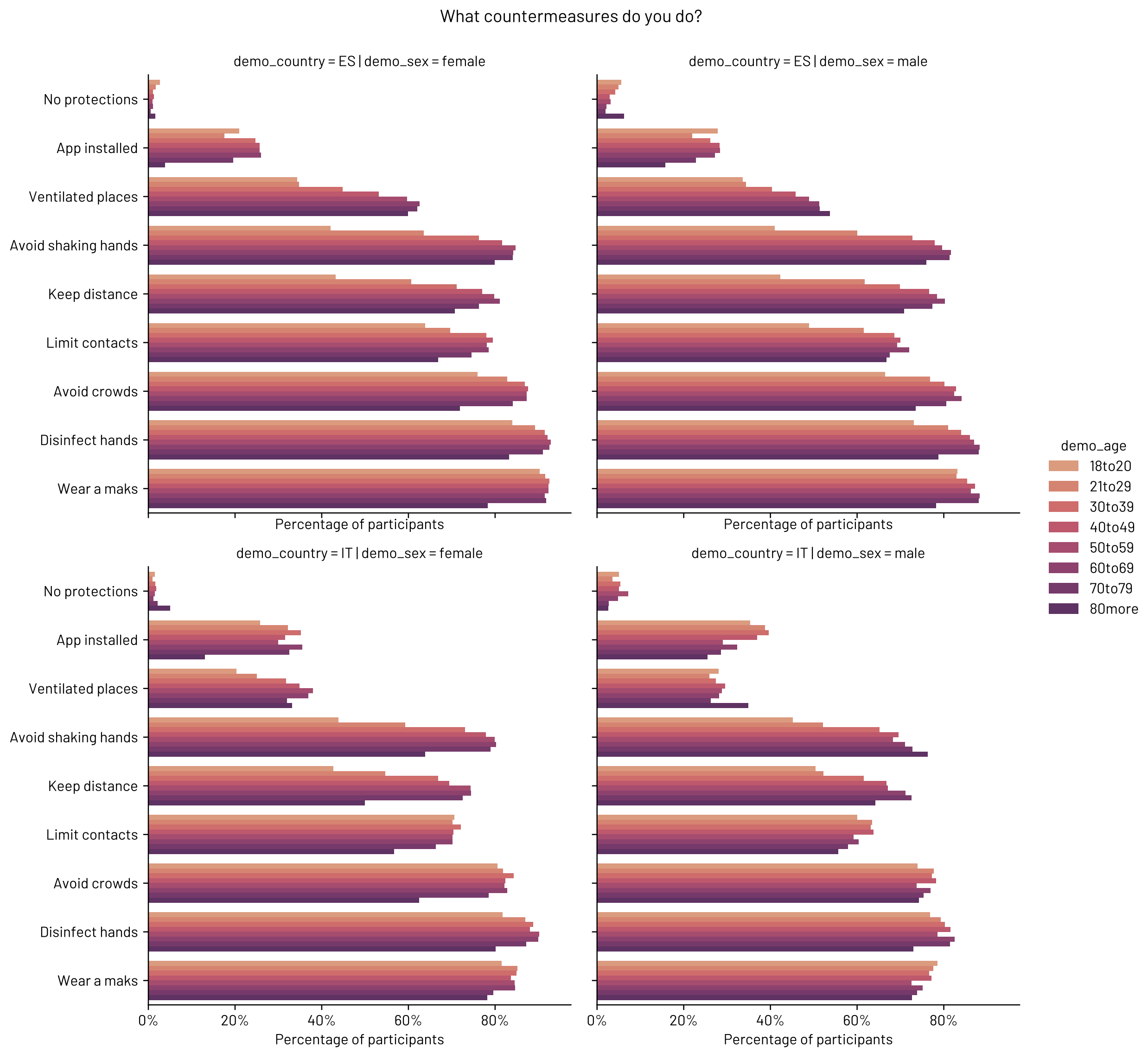

FIG. S16. Individual protection measures per country and demographic group during the Phase I - *new normality*.

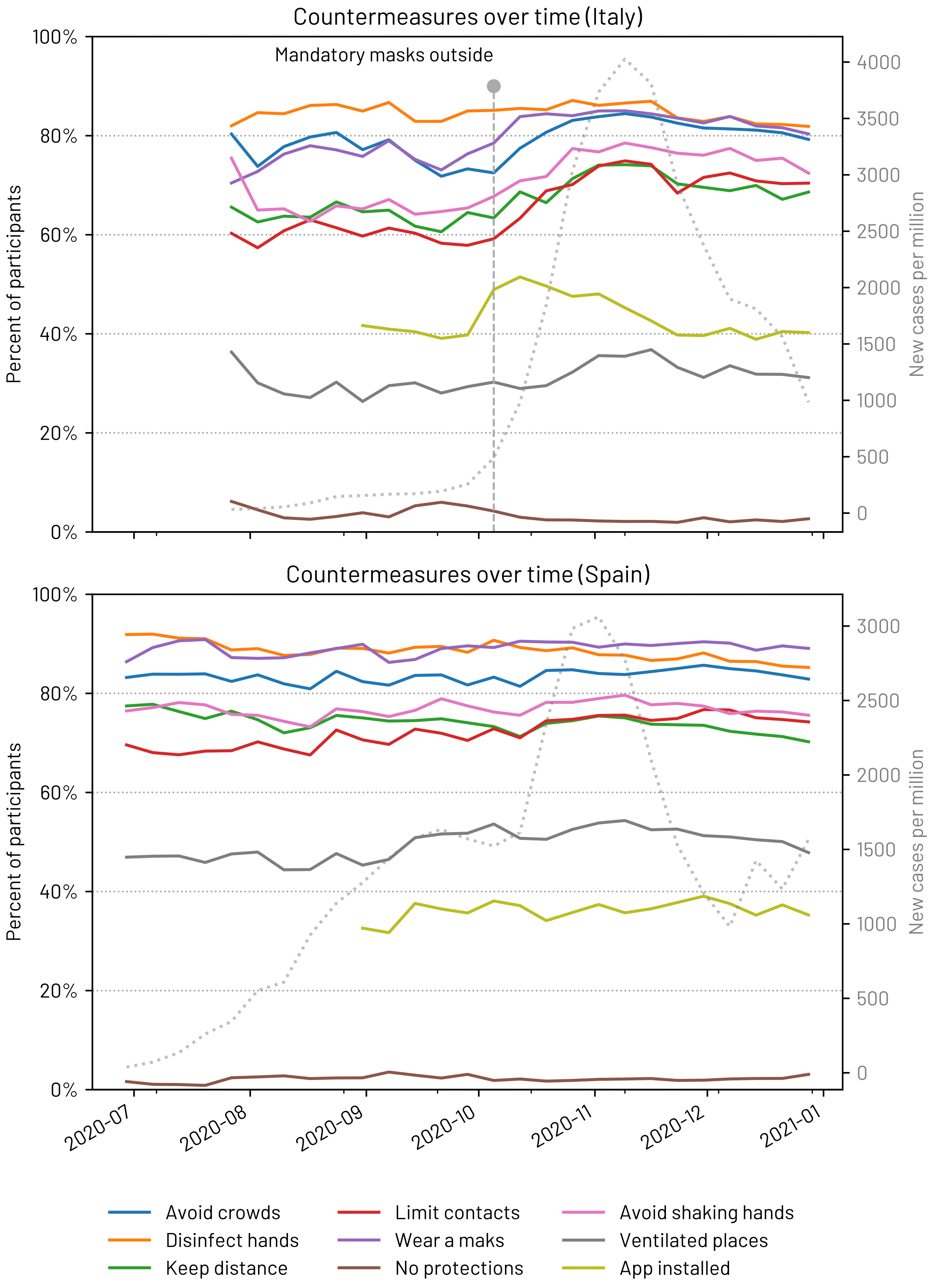

FIG. S17. Evolution of the adoption of individual protection measures over time.

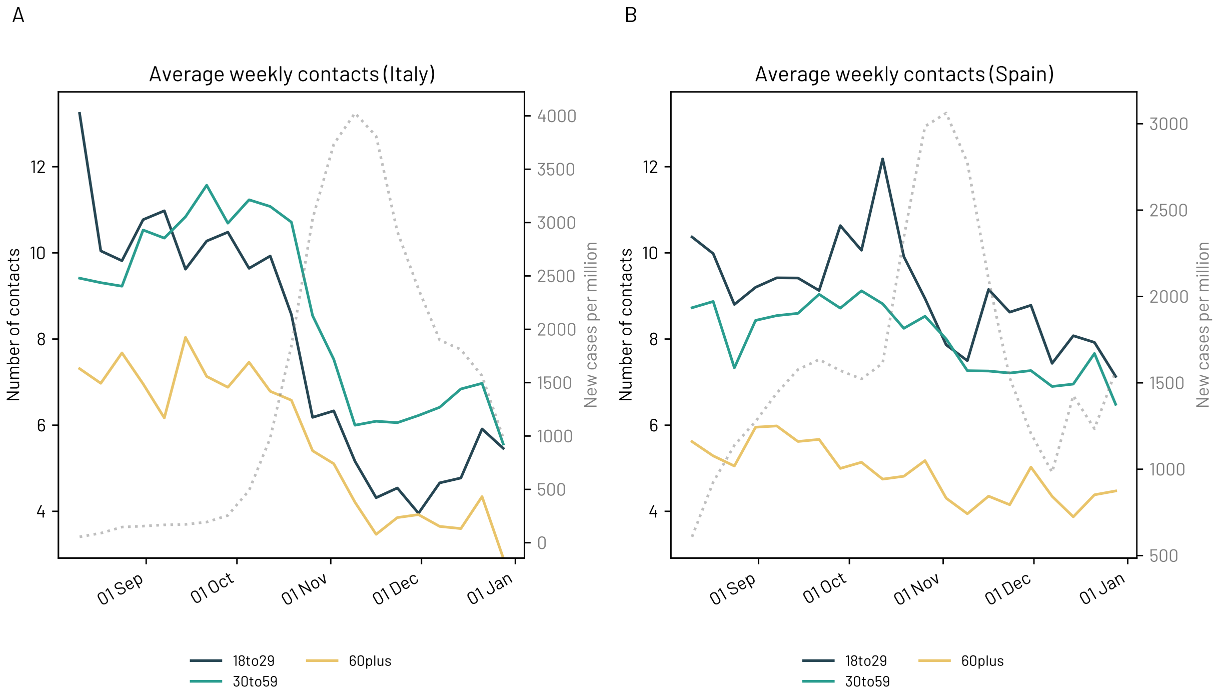

FIG. S18. Average number of weekly close contacts from outside the household per age range (18 to 29, 30 to 59, and 60+ years) in Italy and Spain.

Country	Age range	Period I	Period II	Percentage difference
Spain	18to20	11.52	8.63	-25.15
	21to29	9.07	7.67	-15.49
	30to39	8.96	7.28	-18.71
	40to49	8.62	7.32	-15.10
	50to59	8.39	6.77	-19.33
	60to69	5.58	4.58	-17.96
	70to79	3.76	3.11	-17.50
	80more	5.43	6.96	28.04
Italy	18to20	10.31	4.34	-57.88
	21to29	9.90	5.39	-45.56
	30to39	11.22	6.58	-41.33
	40to49	11.06	6.51	-41.17
	50to59	10.66	6.78	-36.43
	60to69	7.72	4.45	-42.34
	70to79	5.35	2.73	-48.95
	80more	5.90	3.81	-35.52

TABLE S2. Percentage of reduction in the number of close contacts from outside the household between Period I (new normality) and Period II (second wave). We refer with the former with the period before the October 26th in Italy and the November 1st in Spain. We refer with the latter with the period of time after Period I and the end of 2020.

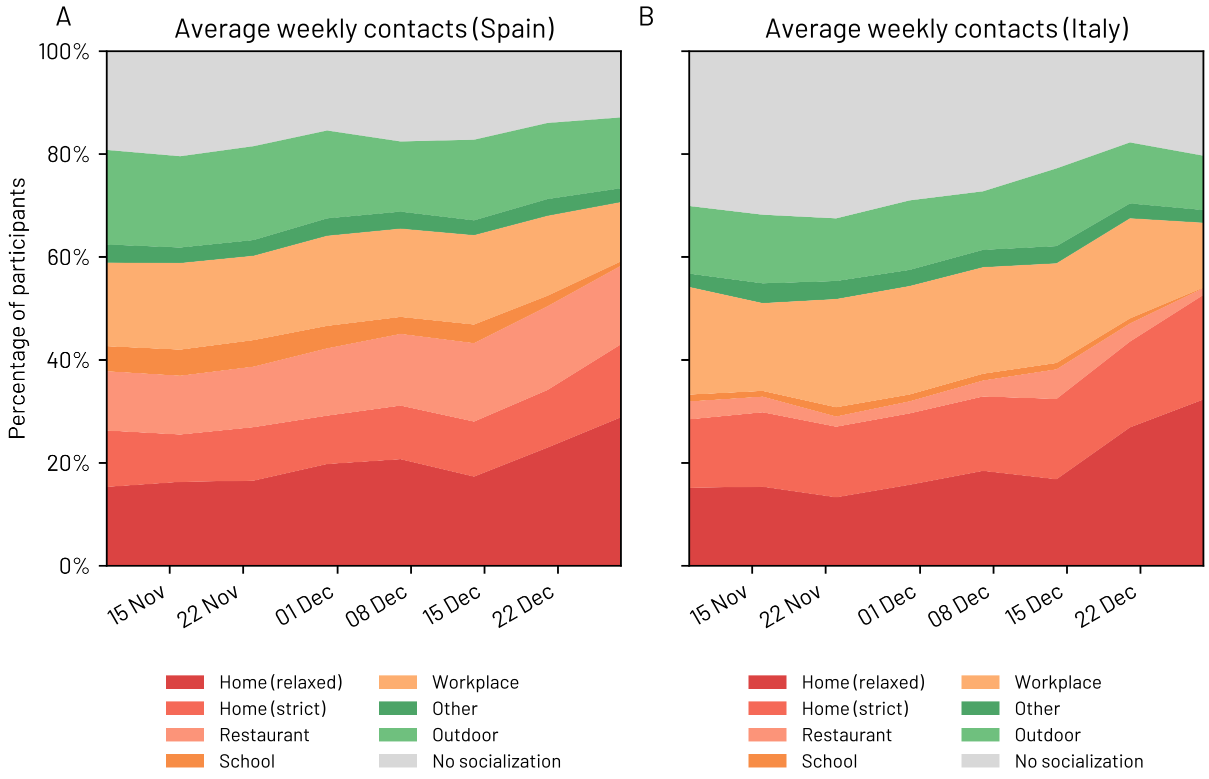

FIG. S19. Breakdown of the main environments of socialisation and their associated risk for Italy and Spain. The colours are chosen to ease the comparison between this Figure and the main paper Figure.

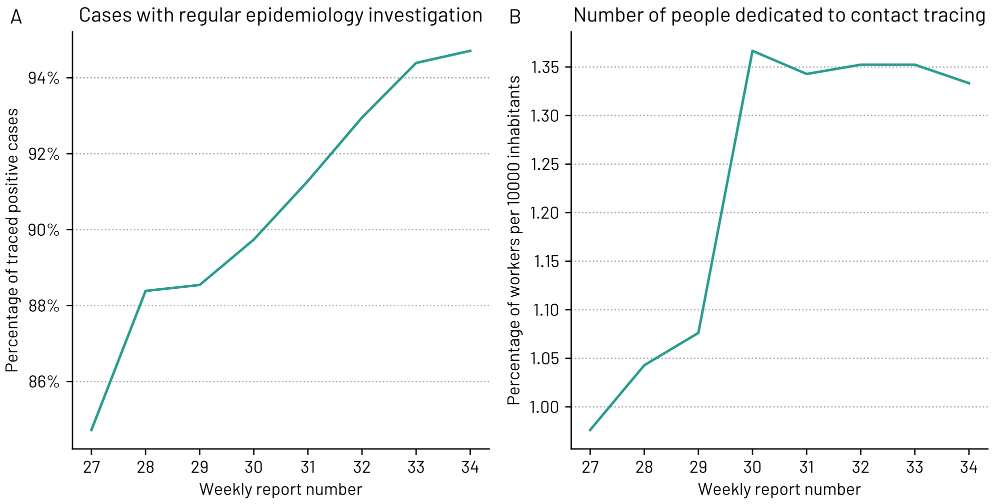

FIG. S20. Official data about contact tracing from the official reports of the Italy Health institute. Data shared by the Italian regions.

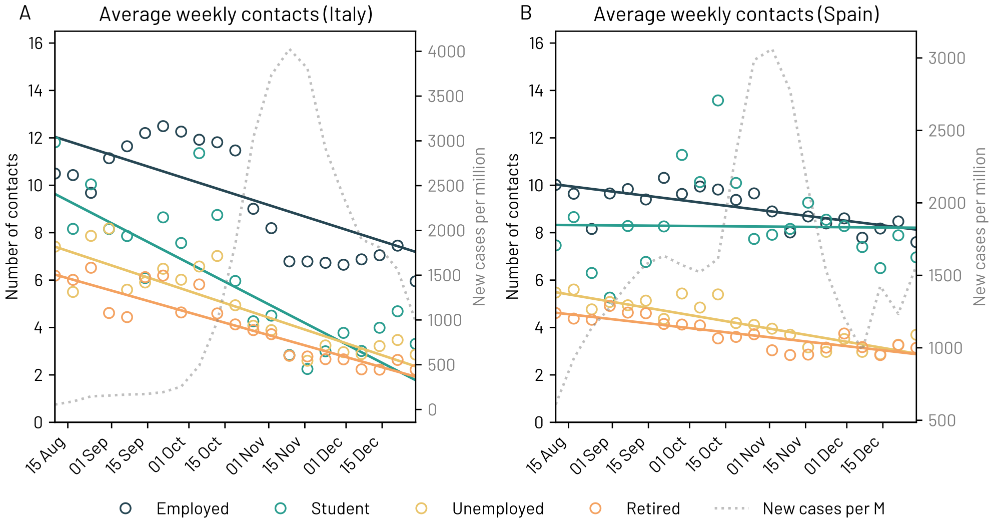

FIG. S21. Trend of the estimated number of weekly close contacts from outside the household together with the number of daily COVID-19 cases (dotted curve) in Italy A) and Spain B). We fitted the average number of weekly close contacts (the dots in the Figure) with a Theil-Sen regression estimator.

S5.2. Trends of weekly close contacts

Figure S21 shows the trends of the weekly close contacts from outside the household fitted through a Theil-Sen regression estimator [16]. More formally, we interpret the number of weekly contacts y as a function of time m and an intercept b: $y = mx + b$. For Italy, we find a $m = -0.24$ and $b = 12.04$ for employed people, $m = -0.39$ and $b = 9.63$ for students, $m = -0.25$ and $b = 7.41$ for unemployed people, and $m = -0.21$ and $b = 6.23$ for retired people.

For Spain, we find a $m = -0.09$ and $b = 10.03$ for employed people, $m = -0.01$ and $b = 8.32$ for students, $m = -0.12$ and $b = 5.48$ for unemployed people, and $m = -0.08$ and $b = 4.623$ for retired people.

S6. ADDITIONAL TABLES

Country	Phase	Age range							
		18to20	21to29	30to39	40to49	50to59	60to69	70to79	80+
Italy	Phase 1	452	2710	3236	3694	4408	4358	1559	155
	Phase 2	1387	3846	4085	4024	4460	3679	1139	103
Spain	Phase 1	1221	6808	11745	14258	15306	10783	3097	218
	Phase 2	3502	5106	5283	5742	6064	4539	1247	90

TABLE S3. Number of answers per country, Phase and age group.

Country	Work type	pre	post	change
ES	Administrative	19.52	14.16	-27.46
	Commercial	4.02	4.23	5.34
	Communications	53.23	48.64	-8.63
	Construction	4.83	2.37	-50.92
	Education	27.85	6.70	-75.95
	Entertainment	21.76	18.75	-13.83
	Essential services	2.22	2.19	-1.47
	Finance	34.76	27.22	-21.68
	Food production	2.53	1.61	-36.27
	Health and social	3.98	2.78	-30.06
	Hospitality	1.43	1.23	-13.67
	Manufacturing	5.44	3.15	-42.02
	Other services	13.13	9.48	-27.78
	Public administration	18.40	11.17	-39.28
	Science, Tech, Professional	36.56	30.75	-15.89
	Transportation	5.68	3.55	-37.63
IT	Administrative	19.54	16.11	-17.55
	Commercial	1.82	5.97	228.44
	Communications	49.14	52.75	7.35
	Construction	3.12	9.86	215.57
	Education	24.56	28.77	17.18
	Entertainment	13.73	33.59	144.68
	Essential services	0.92	3.73	307.05
	Finance	31.01	37.78	21.85
	Food production	2.19	6.90	214.22
	Health and social	4.02	3.05	-24.21
	Hospitality	0.71	2.31	226.95
	Manufacturing	8.55	7.21	-15.69
	Other services	16.77	18.86	12.43
	Public administration	21.15	20.84	-1.44
	Science, Tech, Professional	24.04	23.69	-1.47
	Transportation	8.76	9.47	8.16

TABLE S4. Percentage of reported teleworking in Phase I (pre) and after the end of Phase I (post).

Country	pre	post	change
ES	15.38	9.91	-35.58
IT	14.57	16.00	9.80

TABLE S5. Percentage of reported teleworking in Phase I (pre) and after the end of Phase I (post).

Country	Work type	pre	post	change
ES	Administrative	4.50	4.48	-0.51
	Commercial	4.79	4.88	1.79
	Communications	2.97	4.30	44.71
	Construction	5.21	6.64	27.61
	Education	5.60	2.79	-50.11
	Entertainment	16.90	13.70	-18.91
	Essential services	1.47	2.62	78.25
	Finance	1.41	2.80	99.20
	Food production	10.17	10.53	3.55
	Health and social	3.56	4.33	21.63
	Hospitality	14.21	20.18	42.07
	Manufacturing	5.47	4.88	-10.66
	Other services	8.96	9.39	4.88
	Public administration	0.72	1.73	138.98
	Science, Tech, Professional	2.40	2.08	-13.26
	Transportation	6.58	6.63	0.74
IT	Administrative	1.94	3.27	68.85
	Commercial	3.35	5.20	55.07
	Communications	4.79	5.02	4.94
	Construction	5.84	3.17	-45.72
	Education	5.68	3.11	-45.31
	Entertainment	14.40	23.38	62.37
	Essential services	0.88	1.08	23.36
	Finance	1.25	1.04	-16.66
	Food production	3.63	9.06	149.62
	Health and social	2.61	3.49	33.51
	Hospitality	9.13	25.20	175.91
	Manufacturing	1.54	2.80	81.67
	Other services	6.53	9.14	39.84
	Public administration	1.70	2.11	23.80
	Science, Tech, Professional	2.16	2.60	20.69
	Transportation	4.06	6.17	51.81

TABLE S6. Percentage of reported unemployed participants and respondents on unpaid leave in Phase I (pre) and after the end of Phase I (post).

Country	Type	pre	post	change
Spain	Unpaid leave	3.23	1.56	-51.61
	Lost job	8.34	10.74	28.79
Italy	Unpaid leave	2.02	3.54	75.52
	Lost job	5.92	7.76	30.93

TABLE S7. Percentage of unemployed participants and respondents on unpaid leave in Phase I (pre) and after the end of Phase I (post).

[1] J. Van Bavel, K. Baicker, P. Boggio, V. Capraro, A. Cichocka, M. Cikara, M. Crockett, A. Crum, K. Douglas, J. Druckman, J. Drury, O. Dube, N. Ellemers, E. Finkel, J. Fowler, M. Gelfand, S. Han, S. Haslam, J. Jetten, S. Kitayama, D. Mobbs, L. Napper, D. Packer, G. Pennycook, E. Peters, R. Petty, D. Rand, S. Reicher, S. Schnall, A. Shariff, L. Skitka, S. Smith, C. Sunstein, N. Tabri, J. Tucker, S. van der Linden, P. van Lange, K. Weeden, M. Wohl, J. Zaki, S. Zion, and R. Wille, Using social and behavioural science to support covid-19 pandemic response, Nature Human Behaviour **4**, 460 (2020).

[2] The Digital Transformation Scoreboard, https://ec.europa.eu/growth/tools-databases/dem/monitor/sites/default/files/DigitalTransformationScoreboard2018_0.pdf, accessed: 2020-07-01.

[3] Telework in the EU before and after the COVID-19: where we were, where we head to, https://ec.europa.eu/jrc/sites/jrcsh/files/jrc120945_policy_brief_-_covid_and_telework_final.pdf, accessed: 2020-07-01.

[4] V. Galasso, V. Pons, P. Profeta, M. Becher, S. Brouard, and M. Foucault, Gender differences in covid-19 attitudes and behavior: Panel evidence from eight countries, Proceedings of the National Academy of Sciences **117**, 27285 (2020).

[5] R. Inglehart and P. Norris, The developmental theory of the gender gap: Women's and men's voting behavior in global perspective, International Political Science Review **21**, 441 (2000).

[6] R. Croson and U. Gneezy, Gender differences in preferences, Journal of Economic Literature **47**, 448 (2009).

[7] Pandemic fatigue - Reinvigorating the public to prevent COVID-19, https://apps.who.int/iris/bitstream/handle/10665/335820/WHO-EURO-2020-1160-40906-55390-eng.pdf, accessed: 2021-04-20.

[8] DECRETO DEL PRESIDENTE DEL CONSIGLIO DEI MINISTRI 7 ottobre 2020, https://www.gazzettaufficiale.it/eli/id/2020/10/07/20G00144/sg (), accessed: 2021-04-20.

[9] DECRETO DEL PRESIDENTE DEL CONSIGLIO DEI MINISTRI 24 ottobre 2020, https://www.gazzettaufficiale.it/eli/id/2020/10/25/20A05861/sg (), accessed: 2021-04-20.

[10] DECRETO DEL PRESIDENTE DEL CONSIGLIO DEI MINISTRI 13 ottobre 2020, https://www.gazzettaufficiale.it/eli/id/2020/10/13/20A05563/sg (), accessed: 2021-04-20.

[11] DECRETO DEL PRESIDENTE DEL CONSIGLIO DEI MINISTRI 18 ottobre 2020, https://www.gazzettaufficiale.it/eli/id/2020/10/18/20A05727/sg (), accessed: 2021-04-20.

[12] DECRETO DEL PRESIDENTE DEL CONSIGLIO DEI MINISTRI 3 novembre 2020, https://www.gazzettaufficiale.it/eli/id/2020/11/04/20A06109/sg (), accessed: 2021-04-20.

[13] Guía de la 'nueva normalidad', comunidad por comunidad, https://www.eldiario.es/sociedad/nueva-normalidad-comunidad_1_6067038.html (), accessed: 2021-04-20.

[14] Covid: Spain imposes national night-time curfew to curb infections, https://www.bbc.com/news/

world-europe-54682222 (), accessed: 2021-04-20.

[15] W. S. Cleveland, S. J. Devlin, and E. Grosse, Regression by local fitting: methods, properties, and computational algorithms, Journal of econometrics **37**, 87 (1988).

[16] H. Theil, A rank-invariant method of linear and polynomial regression analysis, in *Henri Theil's contributions to economics and econometrics* (Springer, 1992) pp. 345–381.
